# Mechanism‐Guided Design of Highly Efficient Protein Secretion and Lipid Conversion for Biomanufacturing and Biorefining

**DOI:** 10.1002/advs.201801980

**Published:** 2019-05-01

**Authors:** Shangxian Xie, Su Sun, Furong Lin, Muzi Li, Yunqiao Pu, Yanbing Cheng, Bing Xu, Zhihua Liu, Leonardo da Costa Sousa, Bruce E. Dale, Arthur J. Ragauskas, Susie Y. Dai, Joshua S. Yuan

**Affiliations:** ^1^ Synthetic and Systems Biology Innovation Hub and Department of Plant Pathology and Microbiology Texas A&M University College Station TX 77843 USA; ^2^ Joint Institute for Biological Sciences and Biosciences Division Oak Ridge National Laboratory Oak Ridge TN 37831 USA; ^3^ Department of Chemical Engineering and Materials Science Michigan State University East Lansing MI 48824 USA; ^4^ Department of Chemical and Biomolecular Engineering & Department of Forestry, Wildlife, and Fisheries University of Tennessee Knoxville TN 37996 USA; ^5^ State Hygienic Laboratory University of Iowa Coralville IA 52246 USA

**Keywords:** applied microbiology, heterologous protein secretion expression, lignin valorization, lipid biosynthesis, metabolic engineering

## Abstract

Bacterial protein secretion represents a significant challenge in biotechnology, which is essential for the cost‐effective production of therapeutics, enzymes, and other functional proteins. Here, it is demonstrated that proteomics‐guided engineering of transcription, translation, secretion, and folding of ligninolytic laccase balances the process, minimizes the toxicity, and enables efficient heterologous secretion with a total protein yield of 13.7 g L^−1^. The secretory laccase complements the biochemical limits on lignin depolymerization well in *Rhodococcus opacus* PD630. Further proteomics analysis reveals the mechanisms for the oleaginous phenotype of *R. opacus* PD630, where a distinct multiunit fatty acid synthase I drives the carbon partition to storage lipid. The discovery guides the design of efficient lipid conversion from lignin and carbohydrate. The proteomics‐guided integration of laccase‐secretion and lipid production modules enables a high titer in converting lignin‐enriched biorefinery waste to lipid. The fundamental mechanisms, engineering components, and design principle can empower transformative platforms for biomanufacturing and biorefining.

## 1 Introduction

Bacteria remains one of the most attractive hosts for commercial protein production due to the rapid proliferation, economic media, established fermentation processes, and available genetic toolkit.^1^ Secretory protein production is particularly advantageous for biomanufacturing as it reduces the separation cost, avoids endotoxin contamination, and enables the continuous production preferred by industry and regulatory agencies.^2^ *E. coli* has been the most common bacteria host for protein production, yet the secretory production in Gram‐negative bacteria like *E. coli* is inherently challenging due to the existence of both cytoplasmic and outer members.^3^ Gram‐positive bacteria like *Rhodococcus opacus* could be more amenable to developing an effective secretory production system. Despite extensive research, secretory production of heterologous protein in either Gram‐negative or Gram‐positive bacteria remains highly challenging. The challenge is particularly true for the lignin degradation enzymes with high‐redox potential like laccase, as demonstrated by the low secretory protein yield of laccase in either bacteria or fungi (Figure S1, Supporting Information). The challenge is primarily due to the lack of systemic understanding of protein secretion process and limited efforts to design the secretory production with balanced transcription, translation, secretion, and folding. Emerging systems biology approaches offer new strategies to investigate the protein secretion processes to guide the design of efficient secretory production of heterologous proteins. We, therefore, will address the challenge of secretory protein production with proteomics analysis of *R. opacus* PD630 as a model species to design efficient secretion of a ligninolytic enzyme, where the new system will also address another challenge in biotechnology by enabling the better utilization of *R. opacus* for lignin bioconversion.^4^

Lignin utilization represents one of the imminent challenges in biorefining. Lignin bioconversion is a demanding task due to the inherent recalcitrance of the aromatic heteropolymer.^5^, ^6^ Lignin consolidated bioprocessing via simultaneous lignin depolymerization and aromatic catabolism for bioproduct offers a mean for lignin valorization in a single step.^7^ *R. opacus* has been shown to have the potential for lignin conversion, yet the metabolic and biochemical limits for bioconversion are still elusive. In order to reveal these limits, proteomics and cell growth assay were carried out to compare *R. opacus* PD630 grown on glucose or kraft lignin as the carbon sources (Figure S1, Supporting Information). *R. opacus* cells had limited growth on kraft lignin as compared those on glucose (Figures S2 and S3, Supporting Information), which correlated with our previous findings that lignin depolymerization was necessary for the appreciable growth of *R. opacus* on kraft lignin.^4^, ^8^ The results also corroborated with the secretome proteomics analysis, where no known ligninolytic enzymes were secreted by *R. opacus* PD630 (Figure S1, Supporting Information). The results indicated that lignin depolymerization might be a barrier for efficient bioconversion by *R. opacus*. Our previous study revealed that laccase could synergize with *R. opacus* PD630 to promote lignin depolymerization during bioconversion.^4^ In this study, we therefore, focused on designing effective laccase secretion in *R. opacus* PD630, both as a model for secretory production of heterologous protein and as a functional module to promote lignin bioconversion. Proteomics analysis was carried out to identify the biochemical limits for lignin conversion, to guide the design of efficient laccase secretion, to decipher the metabolic potential for aromatic catabolism and lipid biosynthesis, to discover the mechanisms for carbon partition regulation, and to develop new processes for lignin valorization. The mechanistic discovery and biodesign principle will have broad impact for biomanufacturing and biorefining.

## 2 Results

### 2.1 Secretome Proteomics‐Guided the Biodesign and Engineering of Efficient Secretory Production of Heterologous Laccase

**Figure 1:**
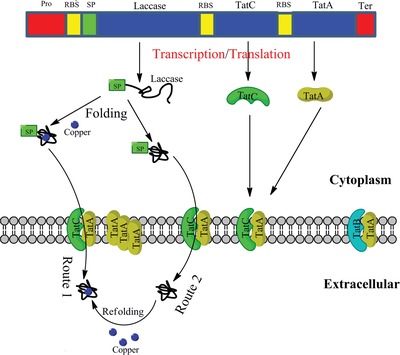
Graphic overview of the biodesign principle to optimize the transcription, translation, secretory machinery, and protein folding.

The challenges in secretory production of heterologous proteins were addressed by redesigning the bacterial secretion machinery at transcriptional, translational, protein secretion and folding levels. In particular, in order to achieve efficient production of extracellular laccase for lignin depolymerization, we carried out proteomics‐guided design from four aspects: promoter/RBS (ribosome binding site), signal peptide, membrane protein transporter, and protein folding (**Figure** **1**).

**Figure 2:**
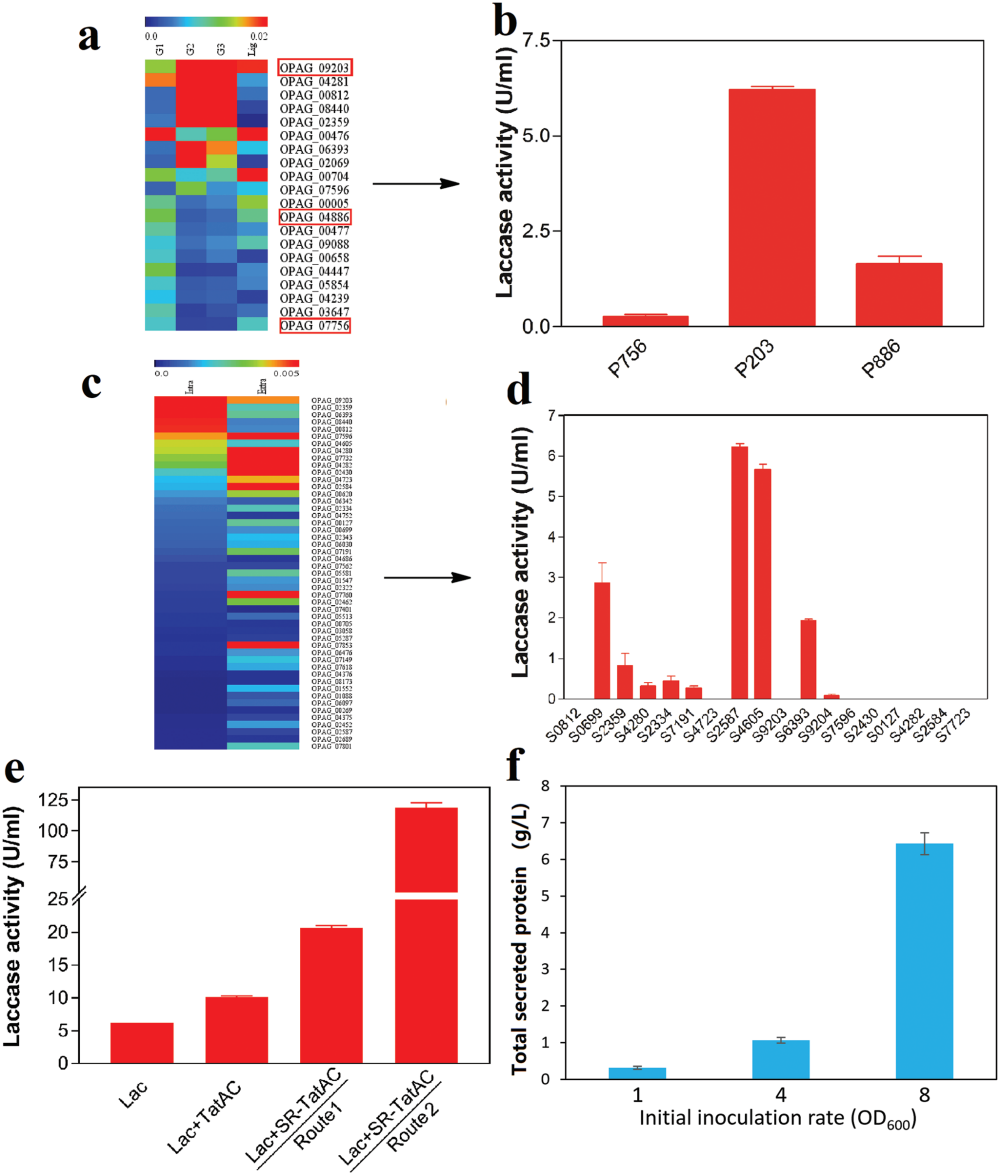
Biodesign of a highly efficient secretory system for heterologous protein production in *R. opacus* PD630. a) The heatmap showed the expression abundance of 20 highly expressed proteins under different conditions. The proteins were sorted (with the highest expressed on the top) by their average abundance in glucose medium from three different bacterial growth phases: log phase (G1), early stationary phase (G2), and late stationary phase (G3). “Lig” represented the proteins from the bacteria grown in kraft lignin as a carbon source. The proteins highlighted by the red square indicated that their promoter and RBS were predicted and used for laccase heterologous expression as shown in (b). b) The extracellular laccase activity for engineered strain with different promoter/RBS derived from the protein highlighted in (a). c) The heatmap showed the comparison of protein abundance between intracellular (Intra) and extracellular (Extra) proteins for the top 50 highly expressed ones with predicted Tat system signal peptides. d) The extracellular laccase activities for engineered strains with laccase integrated with different signal peptides derived from selected proteins in (c). The value showed in the heatmap represented the relative fold change of each protein among different conditions. e) The extracellular laccase activities for an engineered strain with secretory production of laccase and the engineered secretory machinery under different fermentation conditions to optimize protein folding. “Route 1” represented the conventional fermentation strategy with a normal minimum medium, where the expressed laccase would recruit copper and fold into functional protein in the bacterial cell. “Route 2” represented the optimized fermentation strategy, where the laccase was first expressed on a copper‐deficient medium as apoprotein, and then incubated with copper‐containing medium to refold and restore enzyme activity. All aforementioned laccase activities in this figure were measured from the supernatant of fermentation for engineered strains grown on 1% glucose medium after 4 d with 1.4 g L^−1^ NH_4_NO_3_ as a nitrogen source. f) The yield of secreted protein by the engineered strain PD630_La after 7 d of growth on 6% glucose as carbon source and 2.4 g L^−1^ NH_4_NO_3_ as nitrogen source with different initial strain inoculation rates. Three different inoculation rates were used OD_600_ 1.0, 4.0, and 8.0. The protein yield was calculated according to the weight of isolated proteins from the supernatant.

In the first step, the secretory system was optimized for transcription and translation.^9^ Based on the proteomics, five promoters and RBSs for native proteins at different levels of expression were fused with green fluorescent protein (GFP) as a reporter gene to test their strength (Figure S5, Supporting Information). Three of them (P203, P886, and P756) were chosen to drive the expression of the small laccase from *Streptomyces coelicolor* (SLSC, GenBank Number: WP_003972284) (**Figure** **2**a). The native signal peptide for *S. coelicolor* laccase was replaced with the signal peptide S2587 from *R. opacus* PD630. According to enzyme activity, the promoter/RBS P203 derived from one of the most abundant protein OPAG_09203 led to the highest extracellular laccase activity (Figure 2b).

In the second step, we further investigated the secretome of *R. opacus* PD630 and identified several secretory signal peptides to enhance laccase secretion. Considering that SLSC is secreted by the twin‐arginine translocation (Tat) protein transport system in *S. coelicolor*,^10^ we chose the signal peptides from the proteins secreted at a high level via Tat systems based on the secretome proteomics. The Tat system usually transports folded protein and is considered as the primary protein secretion system in many Gram‐positive bacteria.^11^, ^12^ Based on a cluster analysis of secretome and total proteome, eighteen different predicted Tat signal peptides were designed from the proteins enriched in the secretome (Figure 2c). Among the eighteen signal peptides, several secretion peptides including OPAG_7596, OPAG_4282, OPAG_2430, and OPAG_2584 could not drive an efficient secretion of laccase based on enzyme activity screening, even though their corresponding native proteins were expressed abundantly in secretome (Figure 2c). The results highlighted the necessity of designing and screening the proper secretion peptides, as the secretory capacities of native proteins did not correlate with those of the engineered proteins containing the same signal peptides. The phenomena could be due to the inaccurate prediction of secretion peptide or the incompatibility between the signal peptide and target protein, leading to improper protein folding. Among all the peptides screened, SLSC fused with signal peptide S2587 led to the highest enzyme activity and thus potentially highest yield of secretory laccase (Figure 2d).

In the third step, we went beyond the protein design to the secretory system design. Heterologous expression of engineered laccase with S2587 signal peptide at a high level might overload the Tat protein transporters, when protein production rate surpassed the secretion rate. We hypothesized that the capacity of Tat protein secretory system could be another limitation for the efficient secretion of heterologous proteins. The capacity of the Tat secretory system in *R. opacus* PD630 was enhanced by overexpression of the critical components for the Tat transporter machinery, *TatA*, and *TatC* (Figures 1 and 2e).^12^ The overexpression of *TatAC* with their original RBSs (Lac‐TatAC) had increased the extracellular laccase activity by over 60%, validating the hypothesis of limited secretion capacity. In order to further enhance the capacity for secretory system, stronger RBSs derived from the highly expressed proteins in proteomics were integrated with *TatA* and *TatC*. The fusion of RBSs R704 and R756 with *TatA* and *TatC*, respectively (Lac‐SR‐TatAC), resulted in another two‐fold increase of extracellular laccase activity (20.6 U mL^−1^), as compared to that in the *TatAC* overexpressing strains with native RBSs (Figure 2e).

In the fourth step, proper protein folding was enabled by fermentation optimization to complement with the biodesign. Considering the broad substrate specificity of the SLSC, we hypothesized that heterologous overexpression of the enzyme in *R. opacus* PD630 might change the redox environment within the bacterial cell and lead to toxicity, in particular when secretion rate and production rate are not balanced. In order to mitigate the challenge, we designed a two‐step fermentation system. In the first step, laccase was produced as apoprotein by cultivating the engineered strain in a copper‐deficient medium. Without the integration of copper ions, the apoprotein would have no laccase activity, thus would not cause significant changes to the redox environment of the cell. In a second step, the apoprotein was reconstituted to recover laccase activity by adding the copper (Cu^2+^) to the medium. The two‐step fermentation design enabled the extracellular laccase activity to achieve 120 ± 5.49 U mL^−1^ after 4 d of growth in 1% glucose medium, which is 11.7‐fold higher than that cultivated in the copper‐containing medium at the same condition (Figure 2e). The total secretory proteins of the engineered strain were also 7.1‐fold higher than that of the wild type strain (Figure S6, Supporting Information). In order to maximize the potential of the designed secretion system, we further optimized fermentation conditions. We found that the inoculation rate of the strain has a significant impact on the total secreted protein yield. The higher inoculation rate could enable higher secreted protein yield on 6% glucose medium (Figure 2f). The SDS‐PAGE showed that the majority of the secreted protein was targeted protein (Figures S7 and S8, Supporting Information). The higher total secreted protein yield could reach 13.7 ± 2.4 g L^−1^, when the aforementioned engineered strain (Lac‐SR‐TatAC) was grown under the optimized carbon and nitrogen condition (Figure S9, Supporting Information). The maximized laccase yield could provide an economic platform to produce laccase enzyme for the two‐step lignin bioconversion established in our previous study.^8^ The protein yield leapfrogged the current technologies in secretory production of heterologous protein using bacteria system and could have broad applications in biomanufacturing of enzymes, protein therapeutics, and various high‐value proteins. The results highlighted that the systems biology‐guided biodesign at transcriptional, translational, secretory, machinery, and folding levels enabled highly efficient production of secretory proteins in an unprecedented way. The system could not only transform protein biomanufacturing, but also enable lignin depolymerization and conversion.

### 2.2 Proteomics Analysis Revealed the Metabolic Capacity for Aromatic Compound Catabolism

**Figure 3:**
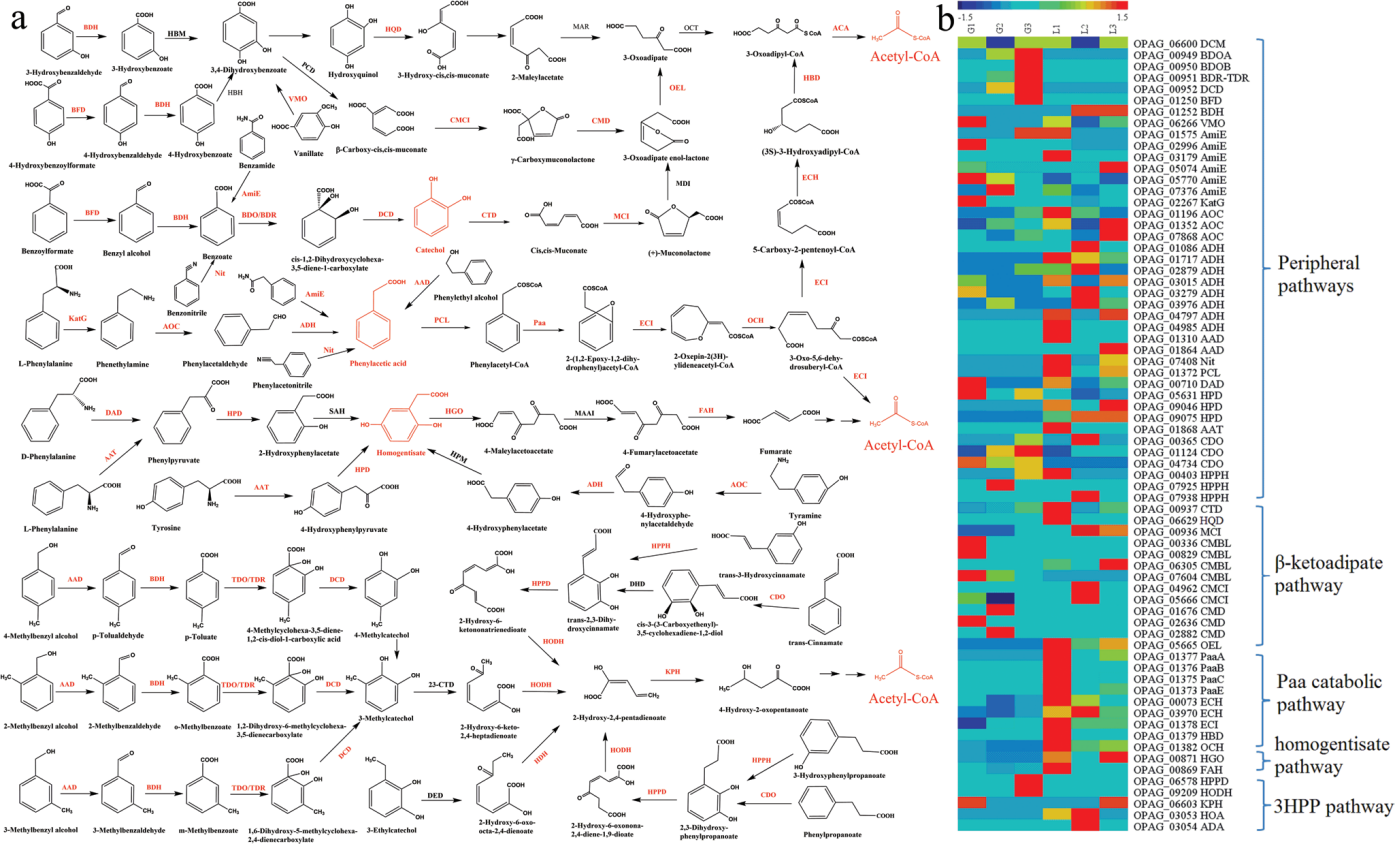
Regulation of catabolic pathways for lignin‐derived aromatic compounds in *R. opacus* PD630 as revealed by proteomics‐guided systems biology. a) The reconstructed catabolism pathways for lignin‐derived aromatic compounds based on proteomics analysis of *R. opacus* PD630. The proteins highlighted in red were detected by the proteomics, and those in black were found from genome but not detected by proteomics in this study. The three aromatic compounds highlighted in red color were the major intermediates for aromatic compound degradation. The metabolites were eventually funneled to acetyl‐CoA for degradation. b) The heatmap of relative expression abundance of differentially expressed proteins involved in aromatic compound catabolism. Each row of the heatmap represents one protein, and each column represented one condition. The data presented in the heatmap was the relative protein abundance normalized by each row (protein) among different conditions. The conditions included the three growth stages of *R. opacus* PD630 grown on 1% glucose (G) or lignin (L) as a sole carbon source: log phase (G1, L1), early stationary phase (G2, L2), and late stationary phase (G3, L3). The proteins presented in this figure were: AAD, aryl‐alcohol dehydrogenase; AAT, aspartate aminotransferase; ACA, acetyl‐CoA acyltransferase; ADA, acetaldehyde dehydrogenase; ADH, aldehyde dehydrogenase (NAD(P)+); AmiE, amidase; AOC, primary‐amine oxidase; BDH, benzaldehyde dehydrogenase (NAD); BDO, benzoate 1,2‐dioxygenase; BDOA, benzoate 1,2‐dioxygenase subunit alpha; BDOB, benzoate 1,2‐dioxygenase subunit beta; BDR, benzoate 1,2‐dioxygenase reductase component; BFD, benzoylformate decarboxylase; CDO, trans‐cinnamate dioxygenase; CMBL, carboxymethylenebutenolidase; CMCI,3‐carboxy‐cis,cis‐muconate cycloisomerase; CMD, 4‐carboxymuconolactone decarboxylase; CMDL, carbon‐monoxide dehydrogenase large subunit; CMDM, carbon‐monoxide dehydrogenase medium subunit; CTD, catechol 1,2‐dioxygenase; 23‐CTD, catechol 2,3‐dioxygenase; DAD, D‐amino‐acid dehydrogenase; DCD, dihydroxycyclohexadiene carboxylate dehydrogenase; DCM, 2,4‐dichlorophenol 6‐monooxygenase; ECH, enoyl‐CoA hydratase; ECI, 2‐(1,2‐epoxy‐1,2‐dihydrophenyl)acetyl‐CoA isomerase; FAH, fumarylacetoacetate hydrolase; HBD, 3‐hydroxybutyryl‐CoA dehydrogenase; HBM, 3‐hydroxybenzoate 4‐monooxygenase; HDH, 2‐hydroxy‐6‐oxo‐octa‐2,4‐dienoate hydrolase; HGO, homogentisate 1,2‐dioxygenase; HOA, 4‐hydroxy 2‐oxovalerate aldolase; HODH, 2‐hydroxy‐6‐oxonona‐2,4‐dienedioate hydrolase; HPD, 4‐hydroxyphenylpyruvate dioxygenase; HPM, 4‐hydroxyphenylacetate 1‐monooxygenase; HPPD, 2,3‐dihydroxyphenylpropionate 1,2‐dioxygenase; HPPH, 3‐(3‐hydroxy‐phenyl)propionate hydroxylase; HQD, Hydroxyquinol 1,2‐dioxygenase; KatG, catalase‐peroxidase; KPH, 2‐keto‐4‐pentenoate hydratase; MAAI, maleylacetoacetate isomerase; MAR, maleylacetate reductase; MCI, muconate cycloisomerase; Nit, nitrilase; OCH, oxepin‐CoA hydrolase; OCT, 3‐oxoadipate CoA‐transferase; OEL, 3‐oxoadipate enol‐lactonase; Paa, ring‐1,2‐phenylacetyl‐CoA epoxidase; PCD, protocatechuate 3,4‐dioxygenase; PCDA, protocatechuate 3,4‐dioxygenase, alpha subunit; PCDB, protocatechuate 3,4‐dioxygenase, beta subunit; PCL, phenylacetate‐CoA ligase; SAH, salicylate hydroxylase; TDO, toluate 1,2‐dioxygenase; TDOA, toluate 1,2‐dioxygenase subunit alpha; TDOB, toluate 1,2‐dioxygenase subunit beta; TDR, toluate 1,2‐dioxygenase reductase component; VMO, vanillate monooxygenase.

Efficient lignin bioconversion involves three steps, including lignin depolymerization, aromatic compound degradation, and bioproduct synthesis.^6^, ^13^ The engineered strain will enable consolidated lignin processing to have kraft lignin depolymerized by secreted laccase for further bioconversion. Once lignin is depolymerized by laccase, the derived aromatic compounds or oligomers can be catabolized by the cell. Proteomics analysis showed the expression of very diverse aromatic compound degradation enzymes and pathways in *R. opacus* PD630 (**Figure** **3**a). Several key enzymes involving the peripheral (upper) degradation pathways for aromatic compounds and aromatic ring‐cleavage (central or lower) pathways were over‐expressed, when *R. opacus* PD630 was grown on the lignin substrate as compared to that on the glucose substrate (Figure 3; Figure S10 and Table S6, Supporting Information).^14^ For aromatic ring‐cleavage, the enzymes in three central aromatic catabolism pathways including β‐ketoadipate pathway, phenylacetate (Paa) catabolic pathway, and homogentisate pathway were significantly over‐expressed in *R. opacus* PD630 grown on lignin as compared to that on glucose (Figure 3b). Among the upregulated enzymes, phenylacetate‐CoA ligase, catechol 1,2‐dioxygenase, and homogentisate 1,2‐dioxygenase were overexpressed by over 50, 15, and 10 folds, respectively, on lignin substrate as compared to glucose (Figure 3b). The results highlighted that phenylacetic acid, catechol, and homogentisate could be the three major central intermediates for the catabolism of lignin‐derived aromatic compounds (Figure 3a). The systemic upregulation of aromatic compound degradation pathways indicated that *R. opacus* PD630 has sufficient capacity to catabolize various lignin‐derived aromatic compounds to central intermediates and eventually to acetyl‐CoA. Our previous study also indicated that *R. opacus* PD630 could efficiently convert the lignin‐derived aromatic compounds into lipids.^8^ The proteomics analysis confirmed the previous discoveries that catabolic enzymes and pathways were coordinated to achieve the conversion of lignin‐derived aromatic compounds to central metabolism. Considering the strong capacity for aromatic compound degradation, we will focus on designing the functional modules for laccase secretion and lipid production to achieve more efficient lignin bioconversion.

### 2.3 Systems Biology Analysis Revealed the Unique Mechanism for High Lipid Production in Oleaginous Bacteria

**Figure 4:**
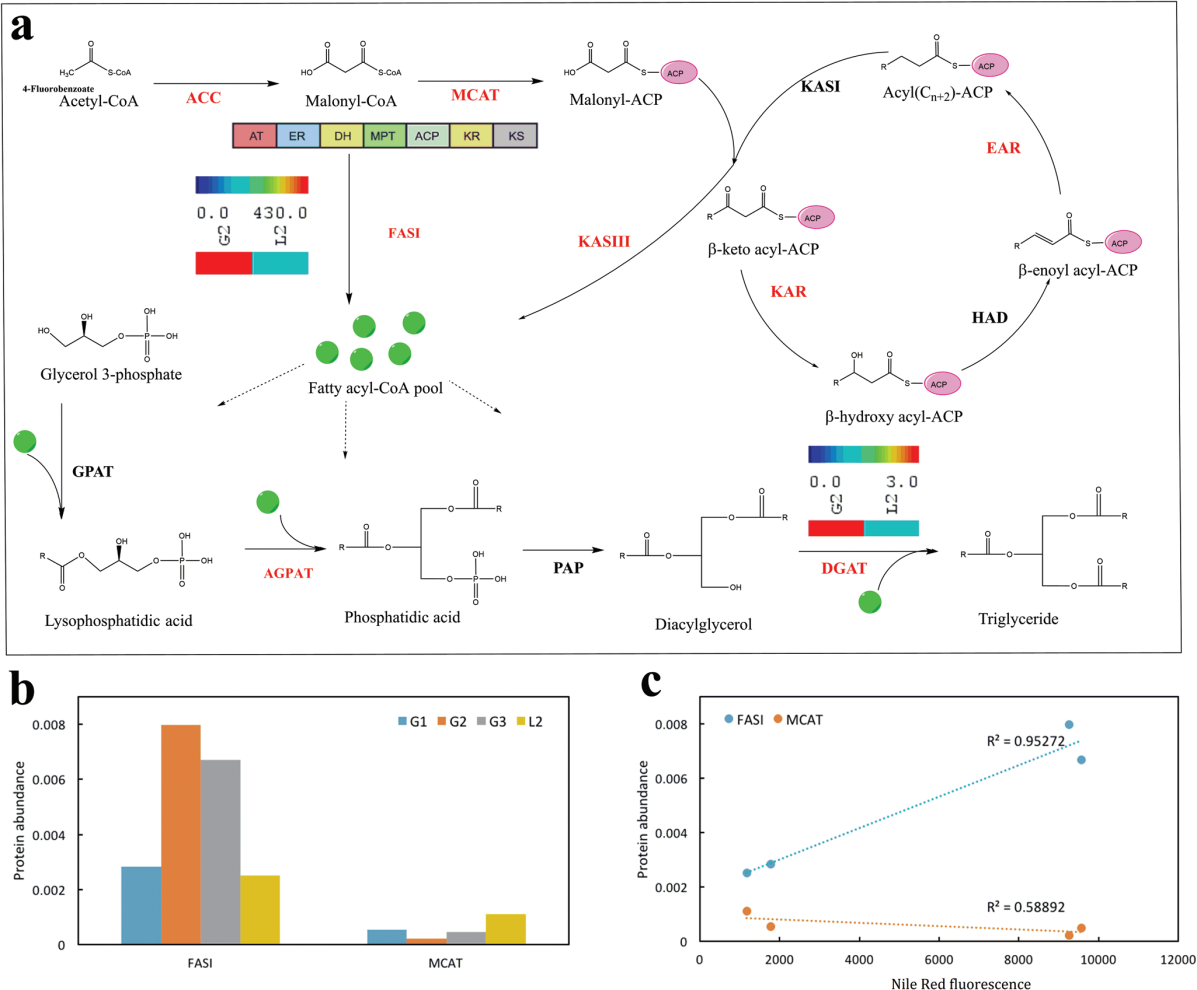
Proteomics‐based systematic analysis of lipid biosynthesis pathway in *R. opacus* PD630. a) The lipid biosynthesis pathway reconstructed based on KEGG. The proteins highlighted in red were detected by the proteomics, and those in black were found from genome but not detected by proteomics in this study. The heatmap showed the enzyme expression abundance between G2 and L2. b) The protein abundance of FASI and MCAT under different conditions. The abundance on *Y*‐axis was presented as their percentage of total normalized peptide counts based on proteomics analysis. c) The correlation efficiency between the protein abundance of FASI or MCAT and lipid accumulation rate. The lipid accumulation was indicated by the increase of Nile Red fluorescence signal under the same condition used for proteomics analysis. The proteins presented in this figure were: AGPAT, 1‐acylglycerol‐3‐phosphate acyltransferase; DGAT, diacylglycerol acyltransferase; EAR, enoyl‐ACP reductase; FASI, type I fatty acid synthase; GPAT, glycerol‐3‐phosphate acyltransferase; HAD, beta‐hydroxyoctanoyl‐acyl carrier protein dehydrase; KAR, 3‐oxoacyl‐[acyl‐carrier‐protein] reductase; KASI, beta‐ketoacyl‐acyl carrier protein synthetase I; KASIII, 3‐oxoacyl‐[acyl‐carrier‐protein] synthase III; MCAT, malonyl CoA‐acyl carrier protein transacylase; PAP, phosphatidic acid phosphatase.

Further pathway analysis revealed the unique regulation of carbon partition for the oleaginous phenotype in *R. opacus* PD630. As aforementioned, the aromatic compounds from lignin funneled to acetyl‐CoA, which could be used as substrates for fatty acid synthesis (**Figure** **4**). Lipid biosynthesis also represents the last step of lignin bioconversion in *R. opacus* PD630. Proteomics analysis under different carbon sources revealed a distinct expression pattern for lipid biosynthesis enzymes, where Type I system (FASI) was significantly upregulated at the stationary phase in glucose medium, when lipid accumulation was stimulated (Figure 4a,b, and Figure S11, Supporting Information). In fact, FASI expression well correlated with the lipid accumulation rate (Figure 4c), indicating FASI as the critical enzyme for the oleaginous phenotype in bacteria. There are two general types of fatty acid biosynthesis systems. Most of bacteria only have Type II system (FASII), which uses discrete and mono‐functional enzymes to carry out decarboxylative Claisen condensation reactions in a step by step way, adding two carbons during every elongation step.^15^ In contrast, FASI exploits single large, multiunit, and multifunctional enzyme to carry out the condensation and elongation to produce palmitic acid (C16) at a higher efficiency. Type I system can further integrate with Type II enzymes to produce a greater variety of fatty acids.^16^, ^17^ FASII is found in plants, archaea, and bacteria, while FASI widely exists in animal and fungal species.^17^ FASI also accounts for the high lipid accumulation in oleaginous fungus. Although most bacteria species do not encode FASI, *rhodococci* is actually among the CMN (Corynebacterium‐Mycobacterium‐Nocardia) group of bacteria encoding FASI in their genome.^18^ *R. opacus* PD630 FASI contains seven functional domains and could catalyze the initiation and elongation reactions of fatty acid synthesis by coordinating with phosphopantetheinyl transferase (PTT), which was expressed in the same operon with *fasI*. Even though both FASI and FASII were expressed in *R. opacus* PD630, only FASI expression showed a high correlation with lipid accumulation rate (Figure 4c). These results suggested that the highly efficient FASI enzyme might account for the accumulation of high lipid content by channeling acetyl‐CoA to fatty acid in oleaginous bacteria like *R. opacus* PD630.

**Figure 5:**
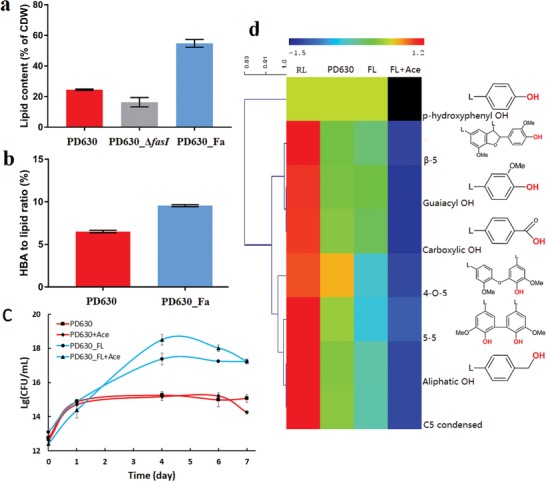
The lipid production and lignin conversion by engineered *R. opacus* PD630. a) The comparison of lipid content between the engineered strain PD630_Fa and the control strain grown on 2% glucose as the sole carbon source with 1.4 g L^−1^ NH_4_NO_3_ as nitrogen source after 4 d. b) The comparison of conversion efficiency from HBA (4‐hydroxybenzoic acid) to lipid between PD630_Fa and the control strain, when they were grown on HBA for 4 d. c) The growth curve of engineered strain PD630_FL and control strain on insoluble kraft lignin as a carbon source. The numbers displayed in the figure was the log (base 10) of CFU. “PD630_FL+Ace” and “PD630+Ace” represented the condition when acetosyringone was used as laccase mediator during fermentation of PD630_FL and PD630. d) Cluster analysis of ^31^P‐NMR characterization of lignin functional groups under different fermentation conditions. FL+Ace: fermentation by engineered strain PD630_FL with acetosyringone as laccase mediator; FL: fermentation by engineered strain PD630_FL without laccase mediator; PD630: fermentation by control strain *R. opacus* PD630; RL: raw lignin without bacterial conversion. The value showed in the heatmap represented the relative fold change of each structural group among different conditions.

The fundamental scientific discovery allowed us to design a functional module to enhance lipid accumulation and improve the conversion efficiency from lignin and carbohydrate. Besides FASI, another enzyme upregulated in the triacylglycerol (TAG) synthesis pathway is diacylglycerol acyltransferase (DGAT, gene *atf2*), catalyzing the final step in the biosynthesis of TAG as the primary storage lipid (Figure 4a). We therefore designed a lipid production module by overexpressing both the *fasI* operon and *atf2* gene in *R. opacus* PD630 to partition more carbon into lipid biosynthesis. The resultant PD630_Fa strain showed a significant increase in the lipid production, assumingly by channeling more acetyl‐CoA into fatty acid and TAG biosynthesis. PD630_Fa produced lipid at a yield of 2.916 ± 0.24 g L^−1^ and content at 54.76 ± 2.08% DCW (Dry Cell Weight) after 4 d of cultivation in glucose, whilst the wild‐type strain only yielded 0.915 ± 0.03 g L^−1^ lipid and achieved 21.03 ± 0.32% DCW of lipid content at the same condition (**Figure** **5**a, and Figure S12, Supporting Information). Further analysis revealed that the introduction of a lipid biosynthesis module did not change cell biomass yield significantly (Figure S12, Supporting Information). When the *fasI* gene was disrupted, the lipid content of the cell was significantly decreased at the same condition, which only had 16.33 ± 2.15% (Figure 5a). The cell biomass and total lipid yield of the *fasI* mutant strain PD630_Δ*fasI* were also significantly decreased as compared to the wild‐type (Figure S13, Supporting Information). The genetic study solidly proved the critical role of *fasI* in storage lipid production in *R. opacus* PD630. Our study also showed that the overexpression of either *fasI* or *atf* 2 along could not achieve significant increase of the lipid yield in *R. opacus* PD630 (Figure S14, Supporting Information). The lipid yield could only be significantly increased when *fasI* and *atf2* were overexpressed simultaneously. This result suggested that even though the overexpression of *fasI* could drive the carbon flux into fatty acid biosynthesis pathway, and the storage lipid could only be accumulated when the synthesized fatty acid was transformed into TAG.

The higher lipid yield thus primarily comes from the carbon repartition, which further verified that FASI was the driving force for higher lipid accumulation in *R. opacus* PD630. Meanwhile, the conversion efficiency from model aromatic compound 4‐hydroxybenzoic acid (HBA) to lipid was also increased by 52.07 ± 1.63% in PD630_Fa as compared to that in the control strain (Figure 5b). Overall, the results validated the hypothesis that the induction of FASI has evolved as the mechanism for the oleaginous phenotype of *R. opacus* PD630, and the mechanism could be exploited to design a lipid production module to enhance lignin bioconversion in *R. opacus*.

### 2.4 Integration of the Laccase Secretion Module and the Lipid Production Module Enabled a High Lipid Titer for the Bioconversion of Lignin‐Containing Biorefinery Waste

The integration of the laccase secretion module and the lipid production module indeed significantly enhanced bioconversion of lignin‐containing biorefinery waste to lipid. We first evaluated the engineered strain cotransformed with the two functional modules (PD630_FL) for their growth performance on 1% (w/v) insoluble kraft lignin as a carbon source. Considering that the *TatAC* overexpressed with strong RBS had resulted in much longer lag phase and lower cell biomass, we used the *TatAC* with their native RBSs for further integration of the two modules, because bioproduct titer relies on cell biomass. The growth of PD630_FL increased ten folds after 4 d as compared to that of the control strain under the same condition, according to the CFU (Colony Forming Unit) assay (Figure 5c). When acetosyringone (2 × 10^−3^ m) was added to the lignin fermentation medium as an electron mediator for laccase, the CFU of PD630_FL was increased 100 fold (Figure 5c). Proper control has been included to ensure that acetosyringone alone does not lead to significant improvement of *R. opacus* PD630 growth. Overall, the result showed that the control strain carrying empty plasmid reached plateau phase 1 day after inoculation, while the engineered strain PD630_FL continued to grow until the fourth day (Figure 5c). Similar with the cell growth, the laccase activity of strain PD630_FL was increasing until the fourth day and then significantly decreased, which might be caused by either laccase absorption to lignin or the loss of laccase expression module in bacteria (Figure S15, Supporting Information). The results indicated that the laccase secreted by PD630_FL could have depolymerized lignin into aromatic compounds or oligomers at low molecular weight to supply the carbon source for sustainable cell growth.

Lignin degradation and consumption by PD630_FL was investigated by ^31^P‐NMR (Nuclear Magnetic Resonance), GPC (gel permeation chromatography), and semiquantitative Prussian Blue assay. First, most of the functional groups were decreased in lignin converted by both *R. opacus* PD630 and PD630_FL (Figure 5d). In particular, the heatmap of the cluster analysis highlighted that the abundance of —OH groups decreased in laccase producing strain PD630_FL. Moreover, the addition of acetosyringone as electron mediator further decreased these structures in lignin (Figure 5d). For example, PD630_FL with laccase mediator led to the most efficient degradation of β‐5 structure at 81.1%, while the control strain PD630 only degraded 33.9% of β‐5 structure. As to the two most abundant hydroxyl groups in kraft lignin, 40.8% of aliphatic and 26.3% of guaiacyl hydroxyl group were degraded by PD630_FL with laccase mediator, as compared to 15.5% and 8.9% degradation of those in control strain, respectively. PD630_FL with laccase mediator also led to more efficient degradation of other functional groups including 4‐O‐5, 5–5, guaiacyl and carboxylic acid at 18.6–21.7%, while the control strain could only degrade 2.7–6.7% of these functional groups (Figure 5d). Second, the lignin consumption was further confirmed by Prussian Blue assay. The Prussian Blue assay showed that the lignin consumption ratio by the engineered strain PD630_FL was significantly higher than that by the control strain, which is consistent with the increased strain biomass of PD630_FL (Figure S16, Supporting Information). Combining with the decreased uncondensed structure and functional groups, the results suggested that lignin was degraded better by PD630_FL strain and small molecular weight lignin might have been consumed to promote cell growth. Third, GPC analysis confirmed that lignin treated by the engineered strain had increased molecular weight changes due to the consumption of low molecular weight compounds derived from lignin depolymerization (Figure S17, Supporting Information). The results corroborated well with our previous studies that laccase and mediators can promote lignin depolymerization and consumption by bacteria.^8^ The results also highlighted that electron mediator could significantly promote the redox reactions by laccase. The electron mediator might improve the electron transfer rate and facilitate the penetration of redox potential into the lignin macromolecule.^19^ Overall, the synergistic activity of the cell, laccase, and electron mediator enabled the efficient depolymerization of lignin, and the mechanism was then exploited to develop a bioprocess for efficient lignin conversion.

**Figure 6:**
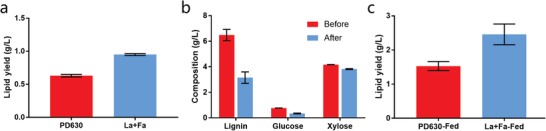
Fermentation of the engineered strain grown on lignin‐containing biorefinery waste from AFEX pretreated corn stover. a) The lipid yield of the consortium of engineered strains by single‐batch fermentation on the lignin‐containing biorefinery waste. PD630: fermentation with the control strain *R. opacus* PD630 on 1.5% biorefinery waste (w/v) for 4 d; La+Fa: cofermentation by engineered strain *R. opacus* PD630_La and PD630_Fa on 1.5% biorefinery waste for 4 d. b) The composition analysis of the lignin‐containing biorefinery waste before and after the single‐batch cofermentation by engineered strain PD630_La and PD630_Fa for 4 d. The composition was analyzed according to the Laboratory Analytical Procedure from the National Renewable Energy Laboratory (Determination of Structural Carbohydrates and Lignin in Biomass). c) The lipid yield of the engineered strain by fed‐batch fermentation on the lignin‐containing biorefinery waste. PD630‐Fed: fed‐batch fermentation of control strain *R. opacus* PD630_Fa on 1.5% lignin waste for 6 d; La+Fa‐Fed: fed‐batch cofermentation of *R. opacus* PD630_La and PD630_Fa on 1.5% lignin waste for 6 d.

Based on the mechanistic study and microorganism engineering, we further designed a bioprocess for the valorization of lignin‐containing biorefinery waste from the AFEX (Ammonia Fiber Expansion) pretreated corn stover. In order to reach the optimal conversion, a cofermentation process was developed to allow each functional module to achieve the best performance. Basically, the strain engineered with laccase secretion module (PD630_La) and the strain engineered with lignin production module (PD630_Fa) were cocultivated for the conversion of biorefinery waste into lipid. After a 4 d fermentation, the lipid yield could research 0.95 ± 0.01 g L^−1^ by the consortium of the engineered strains, as compared to 0.64 ± 0.01 g L^−1^ by control strain (**Figure** **6**a). Lignin utilization by the consortium strains was confirmed by the classic Klason analysis of the lignin waste stream before and after fermentation (Figure 6b). The composition analysis showed that the lignin and xylose were the major components in the lignin waste stream from AFEX pretreated corn stover. The biorefinery waste also contains about 5% glucose. Lignin was significantly consumed after a four‐day fermentation by the consortium strains. The glucose was coutilized, while xylose was largely unused as PD630 has very low capacity in xylose catabolism.^20^ Even higher lipid production at 2.54 ± 0.22 g L^−1^ could be achieved at day six using fed‐batch fermentation with biorefinery waste from AFEX pretreated corn stover, representing the record bioproduct titer from biorefinery waste (Figure 6c).

## 3 Discussion

The study revealed several aspects of fundamental mechanisms regarding protein secretion, lipid biosynthesis, and aromatic utilization in bacteria, which further guided the biodesign of efficient secretory protein production and lignin conversion. For protein secretion, on one side, the results suggested that the key for efficient secretion of heterologous protein lies in the balance of secretion and transcription. On the other side, the secretome revealed the biochemical limits for lignin bioconversion in *R. opacus* PD630, where essentially no known ligninolytic enzyme is secreted, resulting in low lignin depolymerization capacity. The proteomics analysis also unveiled a set of highly expressed and mostly secreted proteins, which can be used to design different components of an efficient secretion system. The high yield of secretory heterologous protein in *R. opacus* PD630 highlighted the path for the effective biodesign of bacteria secretion system, where transcription, translation, secretory machinery and protein folding need to be coordinated to achieve maximized efficiency. In particular, for potential toxic proteins like laccase, it is essential to ensure that the target proteins were secreted once produced, and the target proteins were folded at the proper time to minimize the toxicity. The results also suggested that the mycolic acid and peptidoglycan‐enriched cell wall might not be a significant barrier for protein secretion in *R. opacus* PD630. Further fundamental studies will be needed to understand the exact permeability of the cell wall, and the interaction between signal peptide, target protein, and secretory machinery. Nevertheless, the highly efficient protein secretory system in *R. opacus* offered a transformative platform to manufacture various protein therapeutics, enzymes, and nutraceuticals.

The study also revealed unique mechanisms for lipid accumulation in oleaginous bacteria like *R. opacus*, where the highly efficient FASI enzyme was evolved to channel acetyl‐CoA to fatty acid and TAG biosynthesis. Even though a previous study suggested that FASI was the key enzyme to produce mycolic acids and other fatty acids in the unique cell wall of Mycolata bacteria, the role of FASI in oleaginous phenotype is still elusive.^16^ The correlation between FASI expression and lipid accumulation along with the phenotype in engineered strains well established FASI as the key enzyme driving the carbon partition to storage lipid. The discovery thus enabled the biodesign of *R. opacus* PD630 for high lipid productivity. The designed strain could be broadly used for converting both carbohydrate and lignin to lipid, which could eventually achieve whole biomass conversion. More importantly, the mechanism could also be exploited to channel carbon from lipid biosynthesis to other higher value products, by knocking out the FASI gene and introducing a pathway to shunt the carbon to other bioproducts.

The proteomics study corroborated the genomic analysis in that *R. opacus* PD630 has extensive aromatic catabolism pathways to convert diverse lignin‐derived aromatic compounds into lipid and other bioproducts. This study represented the first proteomics study on *R. opacus* PD630 with lignin as a sole carbon source. Up to eight central aromatic pathways have been identified in rhodococci to date by genome and biochemical studies.^21^ The proteomics analysis identified only three central aromatic pathways significantly upregulated on kraft lignin degradation. These three pathways included phenylacetate (Paa) pathway, β‐ketoadipate pathway, and homogentisate pathway. The results indicated that the aromatic degrading genes were dynamically regulated in response to different aromatic substrates. The proteomics analysis of aromatic catabolism pathways could lay down the foundation for further design of novel routes to produce high‐value aromatic intermediate compounds from lignin. Even though aromatic compound degradation capacity could be still enhanced, the conversion of lignin‐containing biorefinery waste was more limited by the lignin depolymerization capacity, where the extracellular laccase significantly promoted the cell growth and lignin bioconversion.^4^ The laccase mediator further enhanced the electron transfer, lignin degradation, and conversion.^8^ The integration of these biological and chemical designs enabled a high yield of bioconversion product from biorefinery waste. Our previous study also showed that the bacteria could reach better growth by the two‐step lignin bioconversion process, where the lignin was first depolymerized by laccase enzyme, and then the released small molecular compounds were used for bacterial conversion to lipid.^8^ The highly efficient laccase production system in this study could also be used to provide economical and sustainable laccase enzyme for the two‐step lignin bioconversion. The results also highlighted the potential for the designed strains to be used in complete biomass conversion for both carbohydrate and lignin. Besides the fundamental mechanisms, the research thus provided a set of potentially disruptive technologies and synthetic biology toolkits for biomanufacturing and biorefining.

## 4 Experimental Section

*Strain, Plasmids, and Transformation: Rhodococcus opacus* PD630 (DSMZ 44193) was purchased from the German Collection of Microorganisms and Cell Cultures. GenScript synthesized the DNA of small laccase from *Streptomyces coelicolor* with codon optimization according to the codon usage table of *R. opacus* PD630. The *E. coli*‐*Rhodococcus* shuttle vector pBSNC9031 and pT2 were constructed by the authors as described in details in the Supporting Information. Plasmids were introduced into *R. opacus* PD630 by electroporation using electroporator, according to the modified protocol from a previous publication.^22^ The details were described in the Supporting Information. The strains and plasmids used in this study were listed in Table S4 (Supporting Information).

*Total Protein Extraction and MudPIT‐Based Shot‐Gun Proteomics*: For the intracellular proteomics, the total protein was extracted from the bacteria grown on 1% glucose or 1% insoluble kraft lignin as carbon source at different growth stages including middle log phase, early stationary phase, and middle stationary phase, as shown in Figure S2 (Supporting Information). The strain was lysed by Alkali‐SDS buffer, and the proteins were pelletized by chilled tricholoroacetic acid (TCA). For the secretome proteomics analysis, the total secretory proteins were extracted by TCA from the supernatant of bacterial growth medium containing 1% glucose. The extracted protein pellet was air‐dried and then dissolved in a buffer containing 7 m urea, 2 m thiourea, 40 × 10^−3^ m triszma base, and 1% 3‐(4‐Heptyl)phenyl‐3‐hydroxypropyl dimethylammoniopropanesulfonate (C7BzO). The extracted proteins were analyzed by MudPIT‐based shot‐gun proteomics as described in previous publications.^23^ The detailed methods can be found in the Supporting Information.

*Laccase Activity Assay and Protein Concentration Measurement*: The supernatant of the culture medium was collected by centrifugation and used for laccase activity assay and the measurement of extracellular protein concentration. Laccase activity was determined with 2,2′‐azino‐bis(3‐ethylbenzothiazoline‐6‐sulphonic acid) (ABTS) as the substrate at pH 5.0. The extracellular protein concentration was measured following the manual of Pierce Coomassie (Bradford) Protein Assay Kit (Thermo Fisher Scientific). The total secreted protein yield was calculated by weighting the isolated total proteins from the cultural supernatant with TCA‐acetone method as described in the Supporting Information.

*Bacterial Fermentation and Lipid Extraction*: For proteomics analysis, *R. opacus* PD630 was precultured in minimum medium as in the previous publication^4^ with 0.5% glucose as carbon source at 28 °C to OD_600_ 1.5. The cells were washed and suspended in the same volume of culture medium without a carbon source. 5 mL suspended cells were inoculated in 50 mL of aforementioned minimum medium with 1% of kraft lignin (Sigma‐Aldrich #370959) or 1% glucose as carbon source, and 0.4 g L^−1^ (NH_4_)_2_SO_4_ as the nitrogen source. For secretory laccase production, NH_4_NO_3_ was used as the nitrogen source in the minimum medium with different glucose loading as presented in the manuscript. For the biorefinery lignin waste fermentation, the bacteria were precultured in 2% glucose to OD_600_ 8.0. The cells were washed and concentrated in minimum medium without a carbon source. The bacteria were then inoculated to 1.5% (w/v) lignin‐enrich biorefinery waste in the minimum medium with initial inoculation rate OD_600_ 4.0 for 4 d. The fed‐batch fermentation was carried out by cocultivating the strain PD630_La and PD630_Fa in the lignin medium for 3 d and then transferred to the same fresh medium for additional 3 d of fermentation. 2 × 10^−3^ m acetosyringone was added as laccase mediator during lignin fermentation. The lignin waste was extracted from the AFEX pretreated corn stover^24^ with 1% NaOH at 121 °C for 1 h. All of the fermentation was set up at 28 °C with shaking speed of 200 rpm. The total lipid of *R. opacus* PD630 after lignin fermentation was extracted in the form of fatty acid methyl ester (FAME) according to the method described in the previous study.^8^

*Lignin Characterization by ^31^P NMR*: The changes in functional groups and chemical linkages of lignin by different treatments were analyzed by ^31^P NMR.^8^, ^25^ NMR data were processed using the software of TopSpin 2.1 (Bruker BioSpin) and MestreNova (Mestre Labs) packages.^25^ The detailed methods can be found in the Supporting Information.

## Conflict of Interest

The authors declare no conflict of interest.

## Supporting information

Supplementary
