# Supplementary material for: Mechanism‐Guided Design of Highly Efficient Protein Secretion and Lipid Conversion for Biomanufacturing and Biorefining
Source: Adv Sci (Weinh). 2019 May 1;6(13):1801980. doi: 10.1002/advs.201801980 (PMC6662401; doi:10.1002/advs.201801980)
Supplement: Supplementary file 1 — Supplementary [file ADVS-6-1801980-s001.pdf]

Copyright WILEY-VCH Verlag GmbH & Co. KGaA, 69469 Weinheim, Germany, 2019.

ADVANCED SCIENCE
Open Access

Supporting Information

for *Adv. Sci.,* DOI: 10.1002/advs.201801980

Mechanism-Guided Design of Highly Efficient Protein Secretion and Lipid Conversion for Biomanufacturing and Biorefining

Shangxian Xie, Su Sun, Furong Lin, Muzi Li, Yunqiao Pu, Yanbing Cheng, Bing Xu, Zhihua Liu, Leonardo da Costa Sousa, Bruce E. Dale, Arthur J. Ragauskas, Susie Y. Dai, and Joshua S. Yuan**

Supplementary Information

Mechanism-Guided Design of Highly Efficient Protein Secretion and Lipid Conversion for Biomanufacturing and Biorefining

Shangxian Xie, Su Sun, Furong Lin, Muzi Li, Yunqiao Pu, Yanbing Cheng, Bing Xu, Leonardo da Costa Souse, Bruce E. Dale, Arthur J. Ragauskas, Susie Y. Dai, and Joshua S. Yuan

Supplementary Methods 3

Strain and plasmids 3
Plasmid transformation into *R. opacus* PD630 4
Total protein extraction for proteomics 4
MudPIT based shot-gun proteomics 5
Proteomics data analysis 6
Laccase activity 7
Lignin concentration analysis by Prussian Blue assay 7
Lignin characterization by ^{31}P NMR 8
Total lipid extraction 8

Supplementary Tables and Figures 9

Table S1. The laccase activities from different microbes. 9
Table S2. The promoter/RBSs used in this study 10
Table S3. The sequence of signal peptides used in this study 11
Table S4. The expression profile of proteins in lipid biosynthesis pathway 13
Table S5. The list of the strains and plasmids used in this study 14
Table S6. The expression abundance of proteins involved in aromatic compound catabolism. 16
Figure S1. The overview of proteomics analysis of *R. opacus* PD630 grown on 1% glucose or lignin as carbon source. 19
Figure S2. The growth curve of *R. opacus* PD630 grown on 1% glucose or lignin as carbon source. 20
Figure S3. The total protein of bacteria *R. opacus* PD630 grown in 1% of lignin as carbon source. 21
Figure S4. The map of vector pBSNC9031_La used for laccase secretory production in *R. opacus* PD630 22
Figure S5. The fluorescence intensity of GPF in the engineered strain with different protmoters/RBSs. 23
Figure S6. The comparison of yield for total secretory proteins among the engineered strain PD630_La and control strain PD630 on 1% glucose medium on the fourth day. 24
Figure S7. The comparison of total secreted proteins between the engineered strain PD630_La and wild type PD630 on SDS-PAGE. 25
Figure S8. The comparison of total secreted proteins in the culture supernatant between the engineered strain PD630_La and control strains on SDS-PAGE. 26
Figure S9. The yield of secretory protein by the engineered strain PD630_La after 7-days of growth on media with different carbon and nitrogen concentrations. 27

Figure S10. Hierarchical cluster of differentially expressed proteins involved in aromatic compound catabolism. 28
Figure S11. The lipid accumulation of *R. opacus* PD630 grown on 1% glucose. 29
Figure S12. The comparison of lipid yield (left) and cell biomass yield (right) between the engineered strain PD630_Fa and control strain. 30
Figure S13. The comparison of cell biomass yield (left) and lipid yield (right) between the *fasI* mutant strain PD630_ΔfasI and wild type PD630. 31
Figure S14. The lipid content of the strain with overexpression of *fasI* and *atf2*, respectively. 32
Figure S15. The laccase activity of co-transformed strain PD630_FL during fermentation on insoluble alkali lignin. 33
Figure S16. The lignin consumption ratio between the engineered strain PD630_FL and control strain. 34
Figure S17. The molecular weight analysis of the lignin by GPC. 35
Reference 36

Supplementary Methods

Strain and plasmids

Rhodococcus opacus PD630 (DSMZ 44193) was purchased from the German Collection of Microorganisms and Cell Cultures. The sequence of small laccase from *Streptomyces coelicolor* was synthesized by GenScript with codon optimization according to the codon usage table of *R. opacus* PD630. The *E. coli-Rhodococcus* shuttle vector pBSNC9031 was constructed by integrating pBluescript SK with thiostrepton resistance gene tsr from *Streptomyces azureus* and synthesized pNC903 sequence (by GenScript), which contained the *Rhodococcus* plasmid replication origin.[1] The shuttle vector was used for establishing the secretory heterologous expression system and the overexpression of fatty acid synthase. For the construction of the engineered strain PD360_FL, the laccase secretion module and fatty acid biosynthesis module were co-transformed. The optimized laccase secretion modules was expressed in the *Escherichia coli-Rhodococcus* shuttle vector pT2, which contained the *Rhodococcus* plasmid replication origin from pTip-RT1[2], *E.coli* plasmid replication origin, and apramycin resistance gene from pSET152.[3] The strains and plasmids used in this study were list in Supplementary Table 4. For *fasI* gene disruption, the plasmid pK18mob modified from pK18mobsacB by deleting the *sacB* cassette. The DNA of plasmid pK18mob with the kanamycin cassette was inserted to the *fasI* by homologous recombination with the DNA fragment amplified from *fasI* gene with the primers FASI_2170 (5'-GACATCCACGAGATCGACAAC-3') and FASI_3162 (5'-AACGGTGATGGTGATCTGGAC -3').

Plasmid transformation into *R. opacus* PD630

Plasmids were introduced into *R. opacus* PD630 by electroporation using electroporator according to the modified protocol from a previous publication.[4] To prepare electrocompetent cells of *R. opacus PD630*, *R. opacus PD630* was first pre-cultured overnight in Tryptic Soy Broth (TSB), and 0.1 mL of the pre-culture were added to 10 mL of TSB supplemented with 0.5% (w/v) glycine in a 50-mL Erlenmeyer flask. The cells were cultured at 28°C to an optical density of 0.5 at 600nm, and then were harvested and washed twice with ice-cold HS buffer containing 7 mM HEPES and 252 mM sucrose (pH 7.0). The competent cells were concentrated by 10-fold in ice-cold HS buffer. Immediately before electroporation, 400 µl of competent cells were mixed with DNA (final concentration 0.1–1 µg/mL) and pre-incubated at 40°C for 10 min. The electroporation was performed in electrocuvettes with gaps of 2 mm using the following settings: 2.5 kV/cm, 600 Ω, and 50 µF. Pulsed cells were immediately diluted with 1 mL of TSB and incubated at 28°C for 4 h before they were plated on appropriate selection medium.

Total protein extraction for proteomics

For the intracellular proteomics, the total protein was extracted from the bacteria grown on 1% glucose or 1% insoluble alkali lignin as carbon source at different growth stages including middle log phase, early stationary phase, and middle stationary phase, as shown in Figure S2. The strain was harvested from the 50mL culture medium by centrifuging at 5000 ×g. 5 mL Alkali-SDS buffer (5% SDS; 50 mM Tris-HCl, pH 8.5; 0.15 M NaCl; 0.1 mM EDTA; 1 mM $MgCl_2$; 50 mM Dithiothretiol)[5] was added and boiled for 10 min in a water bath. The clear supernatant of the boiled sample was collected by centrifugation at 3000 ×g for 10 min and decanted into a fresh tube. Chilled 100% tricholoroacetic acid (TCA) was added to a final concentration of 20%. The solution was mixed well and incubated at -20 °C for 4 hours. The

pelletized proteins were collected by centrifuging at 16,000 × g for 30 min at 4 °C. The pellet was harvested and washed twice with 1 mL chilled acetone followed by centrifugation at 16,000 × g for 30 min at 4 °C.

For secretome proteomics, the total secretory proteins were extracted from the supernatant of bacterial growth medium on 1% glucose. The aforementioned TCA and acetone extraction method was used to prepare the extracellular proteins.

The extracted protein pellet was air-dried and then dissolved in a solution buffer containing 7 M urea, 2 M thiourea, 40 mM triszma base, and 1% 3-(4-Heptyl)phenyl-3-hydroxypropyl dimethylammoniopropanesulfonate (C7BzO). The extracted protein was stored at -80°C prior to LC-MS/MS proteomics analysis.

MudPIT based shot-gun proteomics

MudPIT-based shot-gun proteomics was carried out to analyze the extracted protein as described in previous publications.[6, 7] Approximately 100 µg of protein was digested by Trypsin Gold, Mass Spectrometry Grade (Promega, WI, USA) with 1:40 w/w at 37 °C for 24 h. The digested peptides were desalted using a Sep-Pak plus C18 column (Waters Limited, ON, Canada) and then loaded onto a biphasic (strong cation exchange/reversed phase) capillary column using a pressure tank. The 2D back column was composed of 5cm of C18 reverse phase resin (C18-AQ, The Nest Group, Inc, Southborough, MA, USA) and 3 cm of strong cation exchange (SCX) resin PolySULFOETHYL A, (The Nest Group, Inc, Southborough, MA, USA). The back column was then connected to a 15-cm-long 100 um-ID C18 column (packed in house with the same C18 reverse phase in the back column) and sprayed through a SilicaTip (New objective, Inc, Woburn, MA). The experiments of two-dimensional liquid chromatography separation and tandem mass

spectrometry followed the protocols previously described by Washburn *et al.*[8] Basically, before SCX separation, a 1 h RP gradient from 100% Solvent A (95% H2O, 5% ACN, and 0.1% formic acid) to 100% Solvent B (30% H2O, 70% ACN, and 0.1% formic acid) was configured to move peptides from C18 resin to SCX resin in the back column. The SCX LC separation was performed with eleven salt pulses containing increasing concentrations of ammonium acetate. Each salt pulse was followed by a 2 h reverse phase gradient from 100% Solvent A to 60% Solvent B. The LC eluent was directly nanosprayed into a linear ion trap mass spectrometer, Finnigan LTQ (Thermo Fisher Scientific, San Jose, CA). The mass spectrometer was set to the data-dependent acquisition mode, and full mass spectra were recorded on the peptides over a 300-1700 m/z range, followed by five tandem mass (MS/MS) events for the most abundant ions from the first MS analysis. The Xcalibur data system (Thermo Fisher Scientific, San Jose, CA) was used to control the LC-LTQ system and collect the data.

Proteomics data analysis

Tandem mass spectra were extracted from the raw files and converted into the MS2 file. The MS2 file was searched against the *R. opacus PD630* protein database established based on the sequences from previous publication.[9] A ProLuCID algorithm was used to search for data.[10] The validity of peptide/spectrum matches was assessed in DTASelect2.0 using a 0.05 false discovery cutoff, a cross-correlation score (XCorr) larger than 1, and normalized difference in cross-correlation scores (DeltaCN) larger than 0.08. Proteins with more than two peptides were identified as detected and were recorded.[6, 11]

Laccase activity

The supernatant of the culture medium was collected by centrifugation and used for laccase activity and extracellular protein concentration assay. The laccase activity assay was determined with 2,2'-azino-bis(3-ethylbenzothiazoline-6-sulphonic acid) (ABTS) as the substrate. The reaction mixtures contained 250 μL 6 mM ABTS in 50 mM sodium acetate buffer (pH = 5.0) and 50 μL culture supernatant. Oxidation of ABTS was monitored by the increase in A420 (ε = 36,000 M−1 cm−1). One unit of enzyme activity was defined as the amount of enzyme required to oxidize 1 μmol of ABTS per min.

Lignin concentration analysis by Prussian Blue assay

Lignin concentration was measured by Prussian Blue assay as described in our previous study.[12] The pH for the sample was adjusted to 12.5 with 10 M NaOH to completely dissolve the lignin. In order to completely dissolve lignin, the lignin sample was mixed at speed of 180 rpm for 1 h. The total volume was adjusted to 100 mL by adding RM minimum medium. The samples were further diluted to an optimal concentration using ddH_2O to adjust the final absorbance at 700 nm to be within the range of 0.7-1.5. Approximately 1.5mL of the diluted samples was transferred into 2 mL tubes. 100 µL of 8 mM $K_3Fe(CN)_6$ was added into the tube and followed by the immediate addition of 100 µL 0.1 M $FeCl_3$. The samples were mixed thoroughly by shaking the tube for 5 minutes. The samples were transferred to 1 cm cuvette to obtain the absorbance at 700 nm by spectrophotometer, using the ddH_2O sample as blank control. Standard curve was established with the same reagents and known concentration of lignin. All experiments were carried out in triplicate.

Lignin characterization by ^{31}P NMR

For quantitative ^{31}P NMR analysis, 20 mg of lignin was dissolved in 500 μL of pyridine/CDCl3(1.6/1.0 v/v) solvent containing chromium acetylacetonate (relaxation agent) and endo-N-hydroxy-5-norbornene-2,3-dicarboximide (NHND, internal standard), then derivatized with 2-chloro-4,4,5,5-tetramethyl-1,3,2-dioxaphospholane. The spectrum was obtained using an inverse-gated decoupling pulse sequence (Waltz-16), a 90° pulse, and a 25 s pulse delay. 128 scans were accumulated for each sample. NMR data were processed using the software of TopSpin 2.1 (Bruker BioSpin) and MestreNova (Mestre Laboratories) packages.[13]

Total lipid extraction

The total lipid of *R. opacus* PD630 after lignin fermentation was extracted in the form of fatty acid methyl ester (FAME) according to the method described in our previous study.[14] The bacteria cells were harvested by centrifugation at 8000g after lignin fermentation. The collected samples were lyophilized for 24 hours and incubated with 20 mL methanol at 65 °C for 30 min. 1 mL of 10 M KOH was then added, and the reaction was incubated at 65 °C for 2 hours. During the incubation, the cap was loosened to release the pressure every 30 min. After the incubation, the sample was moved out from water bath and cooled down to room temperature. 1 mL of sulfuric acid was then added drop by drop, and the reaction was further incubated at 65 °C for another 2 h. 8 mL of hexane was added and incubated for 5 min to extract the lipid. Shaking during the incubation to ensure the proper mixture. The samples were centrifuged at 3500g for 5 min to separate the reaction into two phases. The hexane phase containing the methylated esters was collected to pre-weighted glass tubes. This hexane extraction step was repeated twice. The hexane was then evaporated under nitrogen gas stream and the lipid yield was calculated according to tube weight change.

Supplementary Tables and Figures

Table S1. The laccase activities from different microbes.

Strain	Laccase Activity	Protein location	Substrate for activity assay	Reference
E. coli BL21(DE3) pLysE	13.3 U/mg	Intracellular	ABTS	[15]
E. coli BL21(DE3)	127.78 U/mg	Intracellular	ABTS	[16]
P. pastoris with pHBM905BDM	41 U/mL	Extracellular	ABTS	[17]
E. coli BL21(DE3)	2.94 U/mg	Intracellular	ABTS	[18]
E. coli.	13 U/mg	Intracellular	ABTS	[19]
P. pastoris strain GS115	5.33 U/mg	Extracellular	ABTS	[20]
E. coli BL21(DE3)	16 U/mL	Intracellular	ABTS	[21]
P. capsici strain Phyc12	84 U/mL	Extracellular	ABTS	[22]
Trametes versicolor	0.080 U/mL	Extracellular	RB5	[23]
Fomes fomentarius	0.080 U/mL	Extracellular	ABTS	[24]
*Ganoderma sp.*En3	206 U/mg	Extracellular	ABTS	[25]
Trametes orientalis	20.67 U/mg	Extracellular	ABTS	[26]
Trametes versicolor	243.86 U/mg	Extracellular	ABTS	[27]
Pleurotus sajor-caju	2.350 U/mL	Extracellular	ABTS	[28]
Pleurotus ostreatus	0.522 U/mL	Extracellular	ABTS	[29]
Trametes versicolor	0.629 U/mL	Extracellular	ABTS	[30]
Cerrena sp.	6.3 U/mL	Extracellular	ABTS	[31]
Trametes trogii BAFC 463	5.740 U/mL	Extracellular	ABTS	[32]
Pleurotus ostreatus	0.452 U/mL	Extracellular	DMP	[33]
Coriolopsis gallica	0.250 U/mL	Extracellular	ABTS	[34]
Trametes versicolor	0.045 U/mL	Extracellular	ABTS	[35]
Trametes sanguineus	67.33 U/mg	Extracellular	ABTS	[36]
Trametes versicolor	0.645 U/mL	Extracellular	ABTS	[37]
Nigrospora sp. CBMAI 1328	0.025 U/mL	Extracellular	ABTS	[38]
Shiraia sp. SUPER-H168	11 U/mL	Extracellular	DMP	[39]
Moniliophthora roreri	0.281 U/mL	Extracellular	ABTS	[40]

Table S2. The promoter/RBSs used in this study

**ID**	**Function**	**sequence**	**Source**
P756	Promoter and RBS	CCGGCGAGAACGACGAACGATCCCACCCAGGCGGACAG CGACACCGCGACCGCCTTCGGGGTGGCATCGGTGGGGG TGGCGCGCAGCGGGGTGACCGCGAACTCCGGACGCGGC GGTACGGGTCGCGGCGGTTCCGGCCGGCGTGGCGGCTG CTCGGGTGTCGTCACGCAGCGATCCTATCGACCTGCAGT ACCGTGGCGGGCCGACGCGCGGGTCCGTGTCGGCGGCC CCGAAGCACGCCGCCGAAACACGGCGCCCGAACAAAAT GTGGGCATCCGGGAACAAATCTCCGCACCCCTCCGTTGA GCCTTACGGCAACATGAGCGTGCAAGACTCAAGTTCGA ATTGACTCCCGACGGTGTCGGAGTGCAAACTTGAGCGGA GGGCGCTCACTAAGCGCCAACATCGCAGTTCAGTAATGA AAAACCTGCCCACAGGACTGCAAAACGAAAGTGAGGAA CACT	Upstream sequence of gene OPAG_07756 in *R. opacus* PD630
P203	Promoter and RBS	TCGTTTGTGTAACGCTGGAACTGTCGGCGACGATGTACG TCGATGATGAGTCATCGTGACGGTGGTCTGGAAGTTCCG TGGAAAACGTTCCGTGAGCGTTTGTCAGGGAAGTTCGTG TGACATACAGTCCACCGAGCGCAGTGTGACTTGGAAGA GGTCCGCTGCCGACCCGGCTTCATGCCCGGGCCGGCAGG TCGCCTGTTCCCCGACGGAATCCGCCGGAGCCGCCTAGT GGCGGAGTCCGCAGACGAAGAGAGTGAGCAGTATTC	Upstream sequence of gene OPAG_09203 in *R. opacus* PD630
P886	Promoter and RBS	GCGAGTTCGGTGCGGACGATCGTCGGCCACCCCGGTCCC GCGCCGAGCCTGCGGTAGCCGGAGCTGCCGACGGGGAC GGCCACCGCTTCGAGGGTGGTGCCGGCCCCGGACGTGG GAAGCGGCGCCGCGCCCGCGTACTGCAACCCCCACGGG CGTGCTTCGAATCCCACTGCCCCGATCGCCCCGAGTCCG GCAAATGTCAGGAATTTACGCCGATTGATGTCAGACACC AGGGGAACCTTAGGTGACGACCCGTGAGACAGGCCGAT ATTCGGCTCGGCGCGGTCTACCACCCCCTTCTCCTTGAG CGTTGGCACTCTCACGTATAGAGTGCCAAGTGGCGCCGA TCGAGCTCCGGCACCCGCGACGACGGGGCTGT	Upstream sequence of gene OPAG_04886 in *R. opacus* PD630
Pbne	Promoter and RBS	TACTCCGGGTACCTGTGCGGGCCGCCGGCGATGGTCGAC GCCGGAGTCAAGGCGTTCAAGCGCCGGCGCATGGCTCCT CGACGCATCTTCCGGGAGAAGTTCACGCCCGCCGCGTGA CGACCGCACCCCGATGCGTACGCCGAGGCCCTCCGAGCC TCGGCGTACGCATGCCGGACACGTCCTAGTCAGTACTCG GTAAATGCCTAGTGTGATGCAGCGCACAGGCTGCAAGA GTGGAATTCATTGCCGGACCCGGGCGCCCTCGCAGGGCG CAAGCCCCCGGAGATCTTGGAGGATCCC	Upstream sequence of gene benA in *R. jostii* RHA1
R704	RBS	GACTAGCGGTCGCCTTGTGTGTCACCCCCAATCCGGAGG ATCACTTCGCA	Upstream sequence of gene OPAG_00704 in

			R. opacus PD630
R756	RBS	AGTAATGAAAAACCTGCCCACAGGACTGCAAAACGAAA GTGAGGAACACT	Upstream sequence of gene OPAG_07756 in *R. opacus* PD630

Table S3. The sequence of signal peptides used in this study

Signal peptide	Sequence	Source in *R. opacus* PD630
S0812	ATGAATCGACGGCACTTCGGACGACGGGTCGCGGCAGGTCTGACCGCTGC AGTAGCAGCGACGATGATGTTCACCGGCGTCGTGTCGGCGCAGCCC	OPAG_00812
S0699	ATGACGTCGCAGCGAAGGAGAACGATGGTCAACCGCACTGCAGCGGGTA GGTACGGGGTGCGGTTTGCGCTCGCGGTCGCCCTGACGGCGGCGATTCCG TGCCTCGGAGTCCAGGCGTCCGCTTCCGCCGATCCG	OPAG_00699
S2359	ATGCAGACAGGGACCAGCAGGGGCATGAAACGACTCGCCGGAGGCGCCG CACTCGCTGCCGCAGCCGCCGCCACCGTAGCCGTGACCATGCCGGCGACG GCGTCCGCAGCAACG	OPAG_02359
S4280	ATGAGCGAGAACCGAAAGACCGGCCTGCGCCGTGGAGCCCGCATTGCCG GCCTTGGTGCCGCTGCGGCTGTAGTCCTCGGCCTGATGTCCACGGGTGCG GCCAACGCCGAC	OPAG_04280
S2334	ATGGTCTCTTCGGGCCACGCAGTGCCGCCTGCTGCTAGAGATGGAGTGTC CTTCGTGAAGCGAACCAGAGCTCTTGCAGCAGCGTCACTCGTCGGCGCGG CCGTGACGTTGATCGCATTCGCCGGCCCGGCCGCCGCGAATCCC	OPAG_02334
S7191	ATGGAACGTCGCGTTTCAGCACGCAAGGTCGCGCATCGACGAAGGATCGC AGGCAGTCTGATCGCGCCCGGCGCGTTGGGGCTGGCGGCGCTGCTCGCGA CCCCGTGGTCGAACCCGGGTTCACCGGCCACGACGGCGACA	OPAG_07191
S4723	ATGGACGGTATGAGCGTGATGCGACGCACGATCGCGGCGGCAACGGTCG GTATCGCGGCGACTTTCGGCCTCTCGGGCACGGCAGCGGCGGAACCG	OPAG_04723
S2587	ATGATTGTCACAGCAACGAAGCCGTCACATGGTTGGTTACGAGGCGTCGT CCGGCTGATGGTTGCCGTGGTGATCCTGCCGCTGGCGTTCGTCCTCGTCGG CGGCGGAACGGCCTCCGCCGACCCG	OPAG_02587
S4605	ATGCACACGTCGTCGAACGAGAGTGGTCATATGGGCAAGTCAGGAATCG GTTTTTCCAGGAACAAGCACTGGTCTTCGCGCGTCGCTGTCGCACTGACC GGGGCCGTCGTGTCCGGAACTGCGCTGGTCGGGGCCGCACAGGCCGCACC	OPAG_04605

	G	
S9204	ATGCCGCACCGTCGACCGAAGCCCTCGATCGTCCTCGGGGCTGTCGCCGCTCTGGCTGTCGCAAGTCCCGTCGCGGTCTACGGCATCAGTAGCGCACCCTCC	OPAG_09204
S7596	ATGCGTACCTCGAGGGCGAGTCGGCGCTGCCGTCTCCGATCGAGACCACCACCGCCCCGCAGCGGTAGCTGT	OPAG_07596
S2430	ATGCACACGTCGTCGAACGAGAGTGGTCATATGGGCAAGTCAGGAATCGGTTTTTCCAGGAACAAGCACTGGTCTTCGCGCGTCGCTGTCGCACTGACCGGGGCCGTCGTGTCCGGAACTGCGCTGGTCGGGGCCGCACAGGCCGCACCG	OPAG_02430
S0127	ATGACCAGACTCCGCCGTGTCGCCTCCCTGGCGATGCCCGCCTTACTCGCTTCTACCTGCACGTTTTTCACGATGACGCCCCCTGTTGCGACGGCGGCCCCG	OPAG_00127
S4282	ATGAGCGAGATCCGTAAGTCAGGCCTGCGCCGTGGGGCCCGCGTTGCCGGCCTCGGTGCTGCCGCGGCCGTAGCCCTCGGCCTGATGTCCACCGGTGCTGCGAATGCCGAC	OPAG_04282
S2584	ATGAGCGGACGCCATCGCAAGCCCACCACCACCGGCCGCACCGTCGCCAAGGTCGCCGTCACCGGCGCCATCATGGGCGTCGCCGGAGCAGCCTTCTCGGGCACCGCGAACGCGGCACCC	OPAG_02584
S7732	ATGCGCCCGCGAGGCAACTGCGAACAAACAAACATCAGAAAGGCCCCCATGCGAAGCTCCATCGCACGTCGTGCCGCCGTGTTCGGCTCCGCCGCGCTGCTGCTCCTCGGCCCGGTCGCCGCCTCCGCTCAGGCC	OPAG_07732

Table S4. The expression profile of proteins in lipid biosynthesis pathway

ID	Fold Change	pValue	Protein Abundance*		Protein Name
			G2	L2	
OPAG_00034T0	1.791822	0.664659	5.666667	2	3-oxoacyl-[acyl-carrier-protein] reductase
OPAG_00138T0	1.692276	0.975718	3	1	wax ester synthase/diacylglycerol acyltransferase
OPAG_00610T0	2.82046	0.293004	3	1.666667	phospholipid/glycerol acyltransferase
OPAG_00905T0	1.84612	0.269861	14.66667	5.333333	3-oxoacyl-[acyl-carrier-protein] reductase
OPAG_01295T0	8.461381	0.021719	1	1.666667	3-oxoacyl-[acyl-carrier-protein] reductase
OPAG_01425T0	8.461381	0.021719	1	1.666667	2-hydroxycyclohexanecarboxyl-CoA dehydrogenase
OPAG_01519T0	1.269207	1.310584	4	1	acetyl-CoA carboxylase carboxyl transferase subunit
OPAG_02340T0	-1.05052	0.576783	16	3	3-oxoacyl-[acyl-carrier-protein] reductase
OPAG_02532T0	3.948645	0.167679	3	2.333333	acetyl-CoA carboxylase carboxyl transferase subunit
OPAG_02648T0	-2.27277	0.039747	50	4.333333	2-hydroxycyclohexanecarboxyl-CoA dehydrogenase
OPAG_02951T0	8.461381	0.021719	1	1.666667	3-oxoacyl-[acyl-carrier-protein] reductase
OPAG_02967T0	8.461381	0.021719	1	1.666667	2-hydroxycyclohexanecarboxyl-CoA dehydrogenase
OPAG_04461T0	3.046097	0.367192	1.666667	1	3-oxoacyl-[acyl-carrier-protein] reductase
OPAG_04782T0	3.807622	0.149043	4	3	acetyl-CoA carboxylase, biotin carboxylase
OPAG_05053T0	3.046097	0.367192	1.666667	1	2-hydroxycyclohexanecarboxyl-CoA dehydrogenase
OPAG_05054T0	3.046097	0.367192	1.666667	1	cis-2,3-dihydrobiphenyl-2,3-diol dehydrogenase
OPAG_05807T0	-1.64144	0.463281	8.333333	1	propionyl-CoA carboxylase beta subunit
OPAG_05810T0	4.351567	0.001439	11.66667	10	malonyl CoA-acyl carrier protein transacylase
OPAG_05898T0	1.015366	1.646296	5	1	1-acylglycerol-3-phosphate acyltransferase
OPAG_06418T0	-1.77276	0.438206	9	1	1-acylglycerol-3-phosphate acyltransferase
OPAG_06656T0	3.046097	0.367192	1.666667	1	3-oxoacyl-[acyl-carrier-protein] reductase
OPAG_06946T0	8.461381	0.021719	1	1.666667	acetyl-CoA carboxylase, biotin carboxylase
OPAG_07212T0	25.38414	0.000119	1	5	bifunctional wax ester synthase:acyl-CoAdiacylglycerol acyltransferase

OPAG_07242T0	3.046097	0.367192	1.666667	1	3-oxoacyl-[acyl-carrier-protein] synthase III
OPAG_07243T0	18.61504	0.000956	1	3.666667	3-oxoacyl-[acyl-carrier-protein] synthase III
OPAG_07257T0	18.61504	0.000956	1	3.666667	diacylglycerol O-acyltransferase
OPAG_08335T0	10.15366	0.015625	1	2	3-oxoacyl-[acyl-carrier-protein] reductase
OPAG_09195T0	-3.6854	1.00E-05	430.3333	23	fatty acid synthase Fas I
OPAG_09292T0	-4.92433	0.04924	25	1	biotin carboxylase

* Protein expression abundance was calculated by PatternLab with ACfold analysis, the non-detected proteins were calculated as 1 to calculated the fold change [41].

Table S5. The list of the strains and plasmids used in this study

Strain or plasmid	Description	Source
**Plasmid**		
pTip-RT1	Derived from pRE8424	Tomohiro Tamura[2]
pSET152	Streptomyces ΦC31 integrase-based vector, apramycin resistance	Mary K. Hondalus[3]
pBSNC9031	*E. coli*-*Rhodococcus* shuttle vector, *Rhodococcus* plasmid replication origin from pNC903, thiostrepton resistance	This study
pT2	*E. coli*-*Rhodococcus* shuttle vector, *Rhodococcus* plasmid replication origin from pTip-RT1, apramycin resistance	This study
pBSNC9031_La	small laccase from *Streptomyces coelicolor* replaced its	This study

	signal peptide with S2587 and TatAC with their native RBS were controlled by the promoter P203 in pBSNC9031	
pBSNC9031_La	The FAS1 operon was controlled by the promoter Pben in pBSNC9031	This study
pT2_La	small laccase from *Streptomyces coelicolor* replaced its signal peptide with S2587 and TatAC with their native RBS were controlled by the promoter P203 in pT2	This study
pK18mob	Modified from pK18mobsacB by deleting the *sacB* cassette	This study
**Strain**		
Rhodococcus opacus PD630 (DSMZ 44193)	Wild type	German Collection of Microorganisms and Cell Cultures
PD630_La	Transformed with vector pBSNC9031_La to overexpress the small laccase with S2587 and Tat transporter component TatA and TatC	This study
PD630_Fa	Transformed with vector pBSNC9031_Fa to overexpress the FASI and PTT	This study
PD630_FL	Transformed with vector pBSNC9031_Fa and pT2_La to overexpress the laccase module and FASI module	This study

Table S6. The expression abundance of proteins involved in aromatic compound catabolism.

Protein ID	Protein Abundance*					
	G1	G2	G3	L1	L2	L3
OPAG_01303	0.00002	8.19E-05	8.19E-05	8.19E-05	0	0
OPAG_00708	0.00002	6.27E-05	6.27E-05	6.27E-05	0	0
OPAG_02067	0.00002	0.000105	0.000105	0.000105	0	0
OPAG_01252	0.00002	0.000192	0.000192	0.000192	0	0
OPAG_01630	0.00002	8.06E-05	8.06E-05	8.06E-05	0	0
OPAG_01291	3.07E-05	3.11E-05	3.11E-05	3.11E-05	0	0
OPAG_06605	3.27E-05	3.04E-05	3.04E-05	3.04E-05	0	0
OPAG_02970	0	0	0.00003	0.00003	0	0
OPAG_02890	0	0	0.00003	0.00003	0	0
OPAG_00845	0	0	3.58E-05	3.58E-05	0	0
OPAG_07042	0	0	4.16E-05	4.16E-05	0	0
OPAG_09173	0.00002	6.16E-05	4.38E-05	4.38E-05	0	0
OPAG_06577	0	0	4.74E-05	4.74E-05	0	0
OPAG_01958	0	0	4.95E-05	4.95E-05	0	0
OPAG_07826	0	0	5.32E-05	5.32E-05	0	0
OPAG_01717	0.00002	5.67E-05	5.53E-05	5.53E-05	0	0
OPAG_01124	0	0	6.11E-05	6.11E-05	0	0
OPAG_04962	0.00002	6.75E-05	6.48E-05	6.48E-05	0	0
OPAG_09075	0	0	7.38E-05	7.38E-05	0	0
OPAG_02879	0	0	8.54E-05	8.54E-05	0	0
OPAG_00652	0	0	0.000159	0.000159	0	0
OPAG_01196	0	0	0.000327	0.000327	0	0
OPAG_00854	0	0	4.74E-05	6.59E-05	0	0
OPAG_08008	0	0	0	6.93E-05	0	0
OPAG_06657	0	0	0	7.84E-05	0	0
OPAG_09152	0	0	0	8.38E-05	0	0
OPAG_01217	0	0	0	0.000108	0	0
OPAG_07376	0	0	0	0.000109	0	0
OPAG_05665	0	0	0	0.000109	0	0
OPAG_08218	0	0	0	0.000119	0	0
OPAG_07912	0	0	0	0.000187	0	0
OPAG_01379	0	0	0	0.000538	0	0
OPAG_06220	0	0	0	0	0.000178	0
OPAG_07938	0	0	0	0	0.000238	0
OPAG_01615	0	0	0	0.000159	0.000238	0
OPAG_02310	0	0	0.00003	0.00003	0.000244	0

OPAG_09229	0	0	0	0	0.000335	0
OPAG_02861	0	0	0.000104	0.000104	0.000405	0
OPAG_01086	0.00002	0.000421	0.000421	0.000421	0.001039	0
OPAG_01914	5.41E-05	1.86E-05	1.84E-05	4.09E-05	0.000111	7.61E-05
OPAG_00336	5.41E-05	1.86E-05	1.84E-05	4.09E-05	0.000111	7.61E-05
OPAG_05038	6.83E-05	3.04E-05	1.84E-05	4.09E-05	0.000111	7.61E-05
OPAG_00353	0.000098	1.86E-05	1.84E-05	4.09E-05	0.000111	7.61E-05
OPAG_09087	0.00022	1.86E-05	1.84E-05	4.09E-05	0.000111	7.61E-05
OPAG_06339	6.69E-05	1.86E-05	0.00003	4.09E-05	0.000111	7.61E-05
OPAG_07604	0.000173	8.21E-05	3.22E-05	4.09E-05	0.000111	7.61E-05
OPAG_07477	4.98E-05	5.69E-05	4.16E-05	4.09E-05	0.000111	7.61E-05
OPAG_04615	8.91E-05	5.02E-05	7.05E-05	4.09E-05	0.000111	7.61E-05
OPAG_04734	0.000134	9.79E-05	0.00011	4.09E-05	0.000111	7.61E-05
OPAG_03683	0.000132	0.000101	0.000134	6.59E-05	0.000111	7.61E-05
OPAG_05292	0.001249	0.000103	0.000124	9.09E-05	0.000111	7.61E-05
OPAG_03181	0.000976	0.000266	0.000175	0.000112	0.000111	7.61E-05
OPAG_02510	6.48E-05	0.000118	0.000173	0.000119	0.000111	7.61E-05
OPAG_02203	4.98E-05	3.04E-05	4.74E-05	0.000123	0.000111	7.61E-05
OPAG_03187	0.001316	5.67E-05	0.000112	0.000157	0.000111	7.61E-05
OPAG_03513	0.00047	5.56E-05	0.000111	0.000173	0.000111	7.61E-05
OPAG_04897	0.0001	0.000038	3.58E-05	6.59E-05	0.000172	7.61E-05
OPAG_05770	5.79E-05	4.33E-05	0.00003	4.09E-05	0.000178	7.61E-05
OPAG_04338	0.000218	0.000105	8.64E-05	9.09E-05	0.000178	7.61E-05
OPAG_03454	0.000569	0.000205	0.000249	0.000341	0.000254	7.61E-05
OPAG_04368	7.64E-05	1.86E-05	4.16E-05	6.95E-05	0.000111	0.000113
OPAG_02996	0.000349	7.96E-05	8.64E-05	0.000162	0.000202	0.000113
OPAG_06603	0	0	0	0	0	0.000114
OPAG_06600	0	0	0.00003	6.95E-05	0	0.000114
OPAG_00941	3.07E-05	0.000038	7.37E-05	7.84E-05	0	0.000114
OPAG_06526	0	0	0.00003	0.000233	0	0.000114
OPAG_00829	0.000275	3.11E-05	0.00003	4.09E-05	0.000111	0.000114
OPAG_03248	0.000215	4.98E-05	0.000135	6.59E-05	0.000111	0.000114
OPAG_02207	5.95E-05	1.86E-05	0.00003	6.95E-05	0.000111	0.000114
OPAG_01741	0	0	0	6.59E-05	0.000178	0.000114
OPAG_05666	0	0	3.22E-05	6.59E-05	0.000272	0.000114
OPAG_03015	0	0	0	7.84E-05	0	0.000132
OPAG_05982	0.000693	0.000117	0.000219	0.000103	0.000111	0.000132
OPAG_05631	0.0002	6.05E-05	0.000143	0.000132	0.000111	0.000132
OPAG_07408	0	0	0	0	0	0.00017
OPAG_03041	9.36E-05	1.86E-05	1.84E-05	0.000132	0.000111	0.00017
OPAG_03045	0.000156	3.11E-05	0.00003	0.000202	0.000178	0.000188

OPAG_05074	0	0	0	0	0	0.000188
OPAG_00864	0.000118	0.000074	0.000144	7.84E-05	0.000111	0.000189
OPAG_03859	0	0	0	6.59E-05	0	0.000191
OPAG_03970	8.55E-05	3.11E-05	3.58E-05	0.000294	0.000868	0.000207
OPAG_06955	0	0	0	0	0	0.000228
OPAG_01375	0	0	3.22E-05	0.000781	0	0.000244
OPAG_06340	0.000366	0.00013	0.000136	0.000127	0.000111	0.000244
OPAG_01373	0	0	0	0.000596	0	0.000244
OPAG_00869	8.93E-05	3.11E-05	0.000039	0.000697	0.000349	0.000244
OPAG_01377	0.00002	0.00016	0.000161	0.000329	0	0.000263
OPAG_00936	0	0	0	0	0.000457	0.000263
OPAG_00546	0.000236	7.46E-05	4.16E-05	0.000296	0.000111	0.000263
OPAG_02568	6.38E-05	4.35E-05	5.64E-05	9.09E-05	0.000244	0.000267
OPAG_04898	0.000847	0.000282	0.000368	0.00031	0.000302	0.000281
OPAG_00073	8.55E-05	3.11E-05	6.11E-05	0.000577	0.000868	0.0003
OPAG_07868	0	0	4.38E-05	7.84E-05	0	0.000301
OPAG_06266	0.000236	4.21E-05	4.95E-05	0.000347	0.000111	0.000337
OPAG_03279	0.000256	5.69E-05	5.53E-05	0.000285	0.001646	0.00034
OPAG_00426	0	0	3.58E-05	3.58E-05	0	0.000347
OPAG_00937	0	0	0	0.000634	0	0.000356
OPAG_07311	0.000337	0.000202	0.000281	0.00044	0.000332	0.000357
OPAG_06519	0.000265	6.81E-05	0.000068	0.000319	0.000202	0.000375
OPAG_00945	3.27E-05	3.62E-05	0.00003	0.000244	0.00041	0.000413
OPAG_09141	0.000574	0.000161	0.000121	0.000477	0.000444	0.000415
OPAG_00710	0.000343	1.86E-05	1.84E-05	0.000615	0.000111	0.00045
OPAG_08214	0.000251	1.86E-05	3.58E-05	0.000655	0.000178	0.000467
OPAG_03805	0.00042	6.74E-05	0.000153	0.000448	0.000206	0.000474
OPAG_01352	0	0	0	0.000208	0	0.000497
OPAG_09096	0.000321	3.15E-05	5.32E-05	0.000746	0.000206	0.000542
OPAG_06121	0	0	0	0.000335	0	0.000546
OPAG_00675	0.000531	0.000123	0.000276	0.000612	0.000172	0.0006
OPAG_00871	0	0	0	0.000407	0.000263	0.000694
OPAG_01085	9.45E-05	9.16E-05	9.32E-05	0.000566	0.000172	0.000696
OPAG_07840	0.001156	0.000185	0.00029	0.000657	0.00071	0.00099
OPAG_01372	0.00002	0.000578	5.32E-05	0.001026	0.000475	0.001048
OPAG_07409	4.54E-05	0.000038	5.64E-05	0.001037	0	0.001048
OPAG_04511	7.99E-05	0.000236	0.000276	0.00103	0.000273	0.001141
OPAG_04895	0.003954	0.000346	0.00052	0.000902	0.000293	0.001182
OPAG_04896	0.001202	0.00027	0.000284	0.00076	0.000302	0.001216
OPAG_01378	5.25E-05	0.000234	0.000177	0.001188	0.001522	0.001331
OPAG_09046	0.00002	4.98E-05	4.98E-05	0.000851	0.000387	0.001578

OPAG_02267	0.006458	0.000666	0.001087	0.002041	0.000429	0.001837
OPAG_01382	0.000211	3.15E-05	4.16E-05	0.004318	0.004984	0.004431
OPAG_04741	7.86E-05	0.000106	7.06E-05	0.008162	0.003063	0.015208

* Protein expression abundance was calculated by PatternLab and the number showed in the table was normalized according to their percentage among total proteins detected.

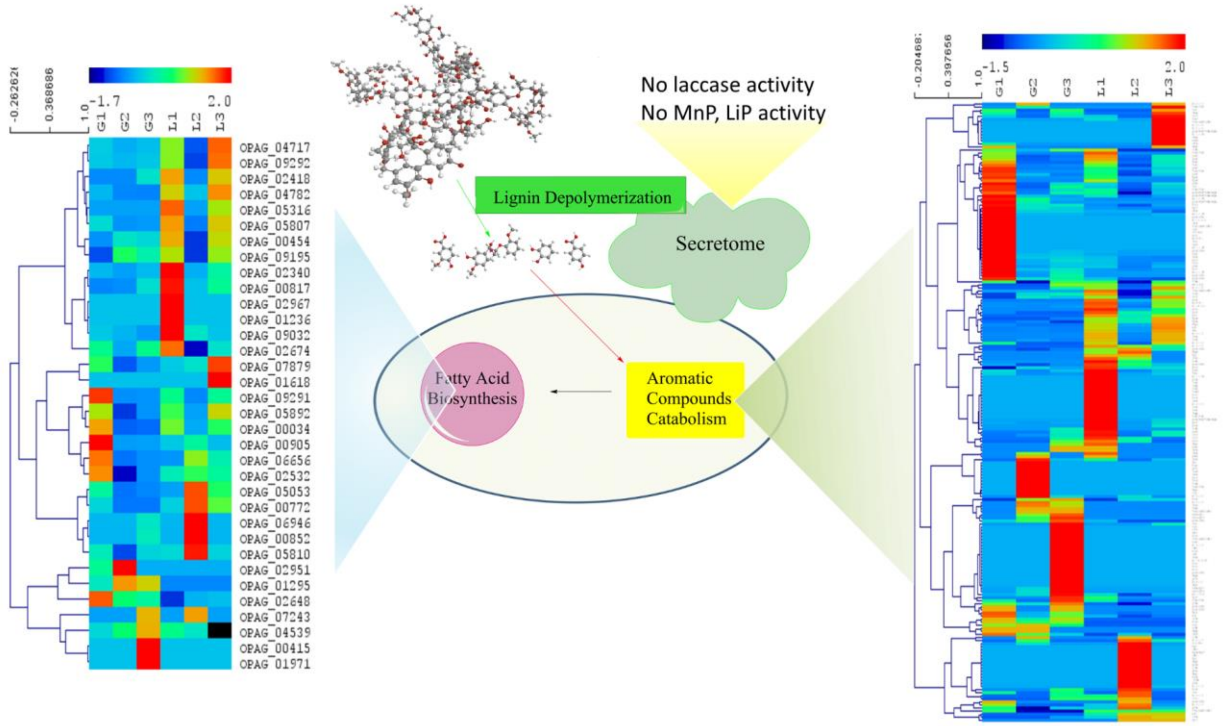

Figure S1. The overview of proteomics analysis of *R. opacus* PD630 grown on 1% glucose or lignin as carbon source. The capacity of lignin depolymerization, aromatic compound catabolism, and lipid biosynthesis were analyzed by proteomics and guided the biodesign of *R. opacus* PD630 for efficient lignin bioconversion into lipid.

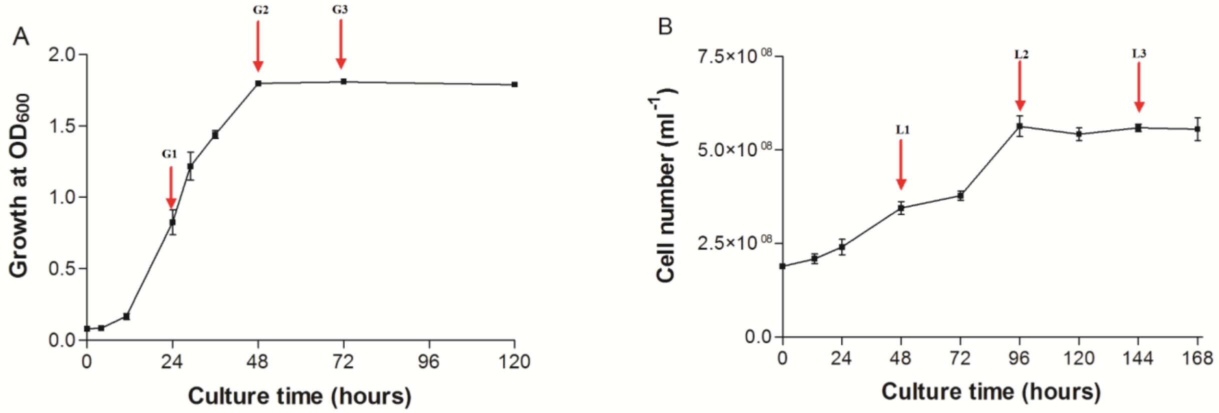

Figure S2. The growth curve of *R. opacus* PD630 grown on 1% glucose or lignin as carbon source. The red arrow indicated the sample collection timing for proteomics analysis. The alkali lignin used in this study was purchased from sigma (Sigma-Aldrich #370959).

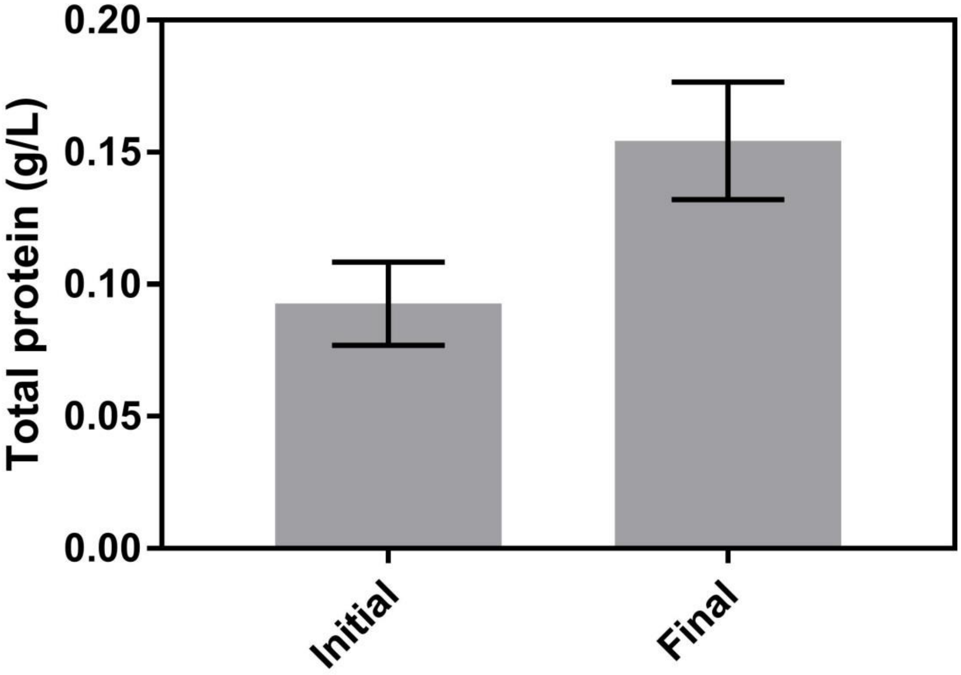

Figure S3. The total protein of bacteria *R. opacus* PD630 grown in 1% of lignin as carbon source. “Initial” represented the total protein extracted from the bacteria at the time inoculating to the lignin medium; “Final” represented the total protein extracted from the bacteria grown in 1% lignin medium for 96 hours, which is the sample indicated as “L2” in Figure S2. The significant total protein increase was observed, which indicated that the increase of the CFU is caused by actual bacterial growth on lignin instead of just bacterial reductive division.

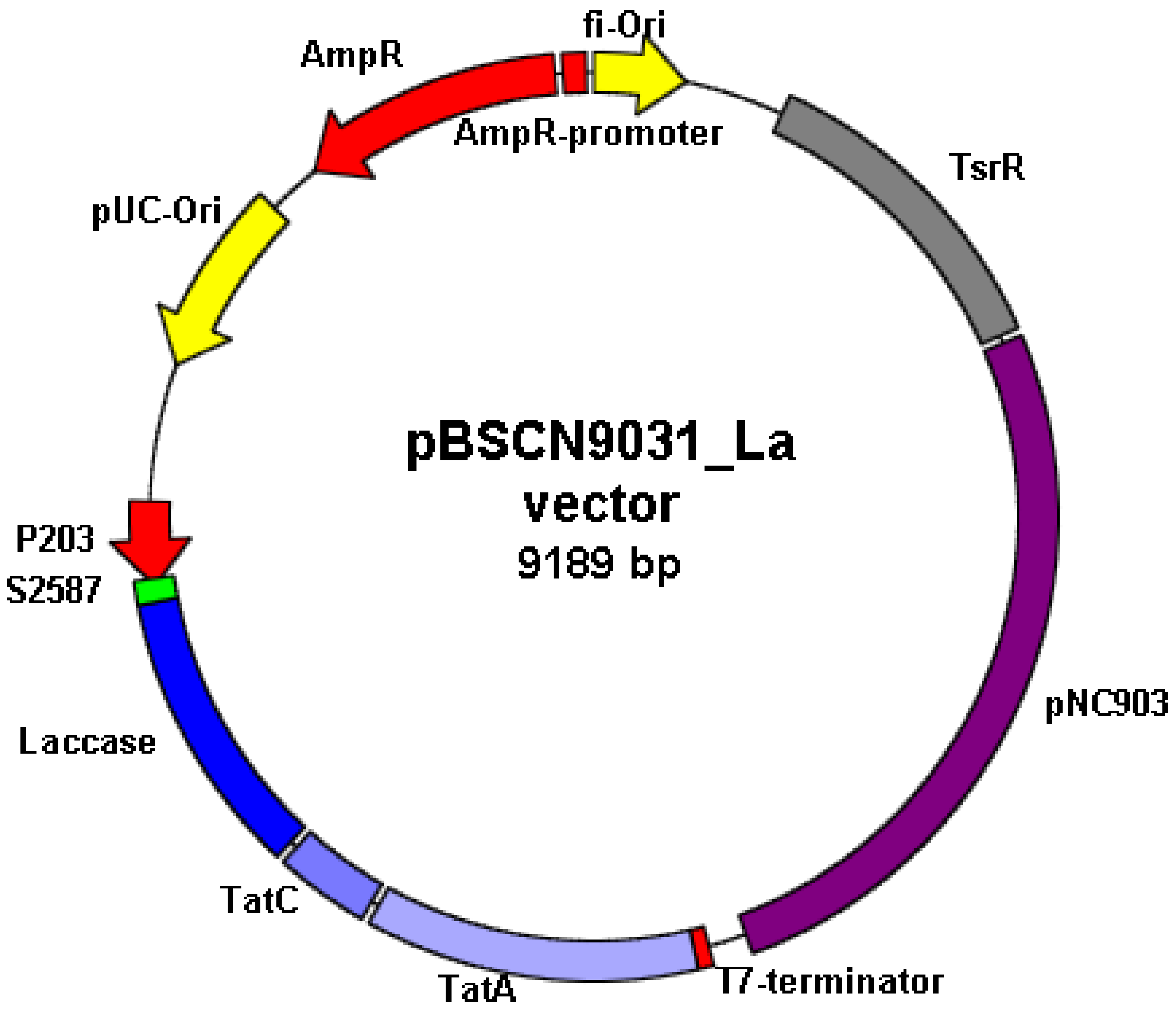

Figure S4. The map of vector pBSNC9031_La used for laccase secretory production in *R. opacus* PD630

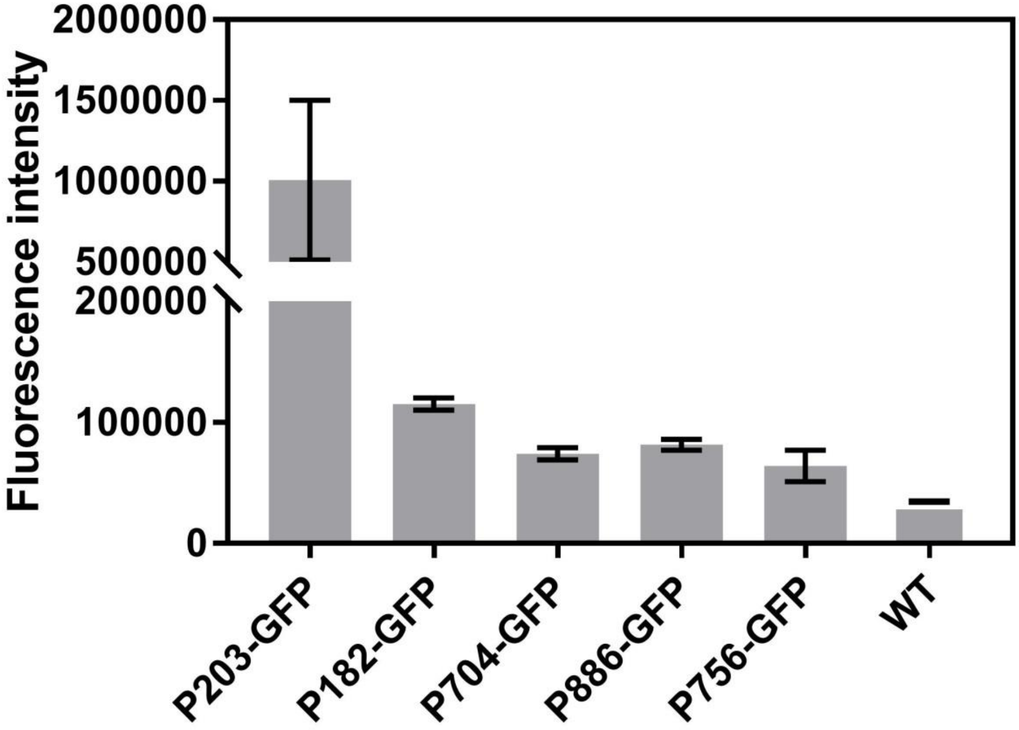

Figure S5. The fluorescence intensity of GPF in the engineered strain with different protmoters/RBSs. The strain engineered with report gene GFP controlled by different promoters/RBSs were grown in 0.5% of glucose medium to measure the promoters/RBSs strength.

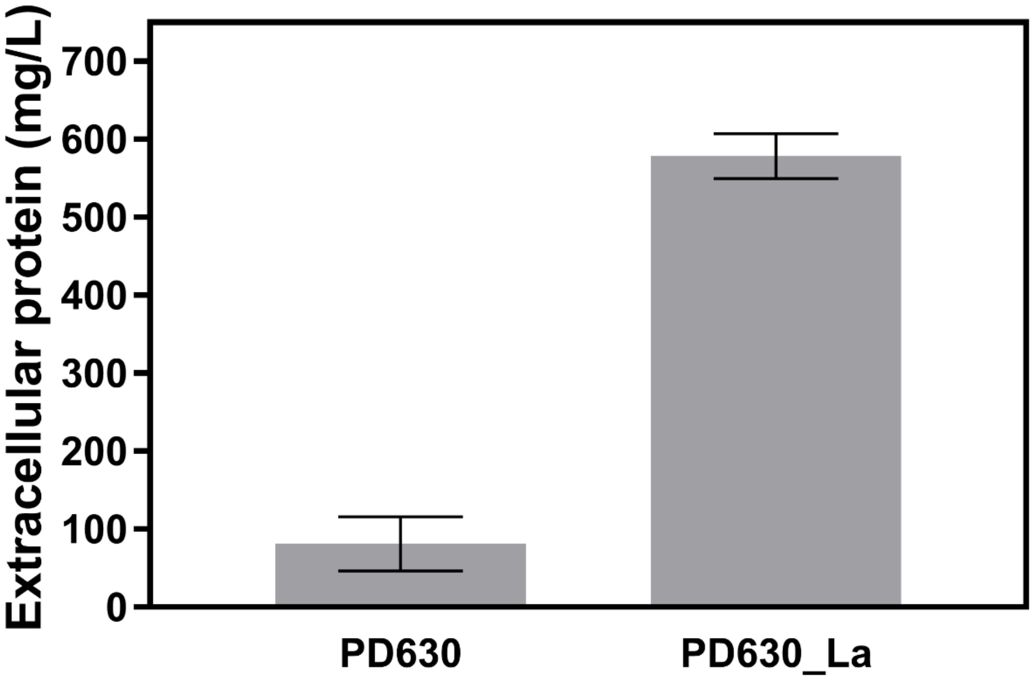

Figure S6. The comparison of yield for total secretory proteins among the engineered strain PD630_La and control strain PD630 on 1% glucose medium on the fourth day.

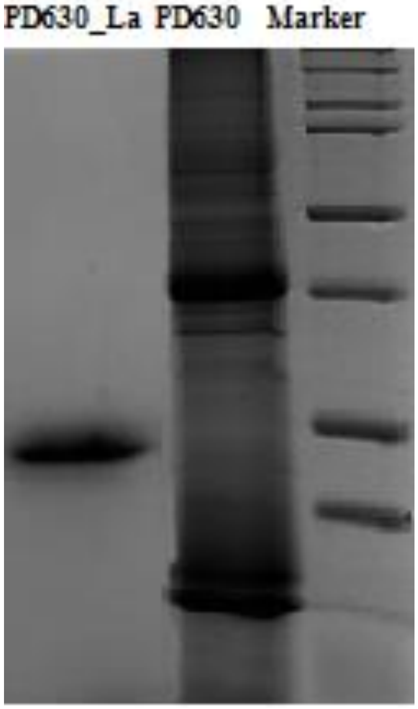

Figure S7. The comparison of total secreted proteins between the engineered strain PD630_La and wild type PD630 on SDS-PAGE. The proteins from the supernatant were collected by TCA method as described in the Methods section, and 50 µg of the concentrated proteins were loaded to the gel.

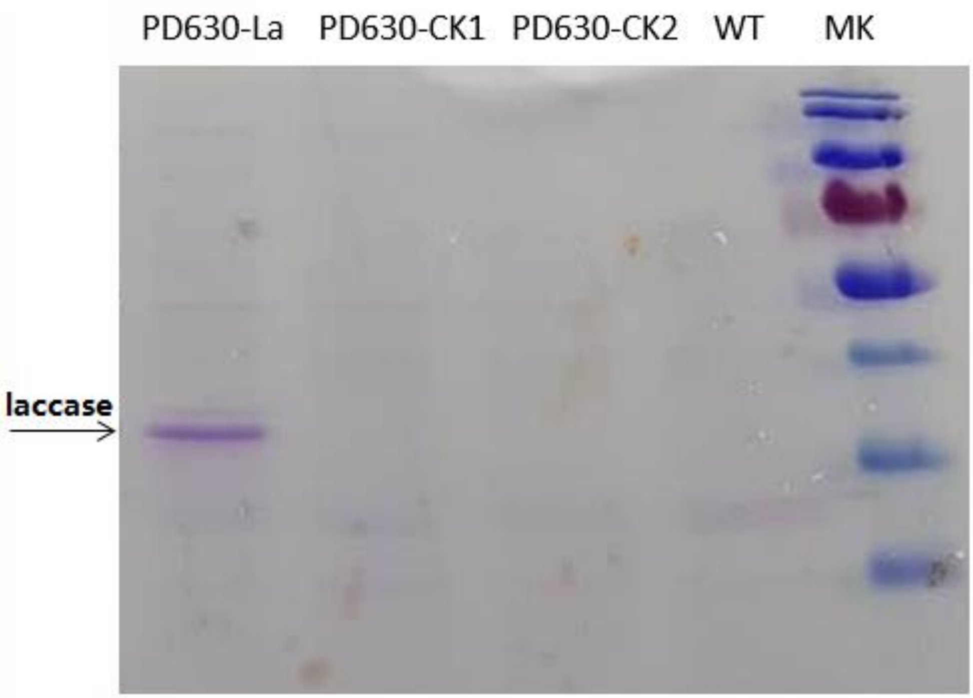

Figure S8. The comparison of total secreted proteins in the culture supernatant between the engineered strain PD630_La and control strains on SDS-PAGE. The strains grown on 2% of glucose for 7 days, and the supernatant was collected by centrifuging to remove the cells. 50 μL of culture supernatant without protein concentration were used to load on the SDS-PAGE. PD630_CK1 was the control strain engineered with the same promoter/RBS and signal peptide with PD630_La, but the laccase was replaced by a protein lunasin which could not be secreted with the S2587 signal peptide; PD630_CK2 was the control strain engineered with the same plasmid but without laccase gene; the WT is the wild type strain of PD630.

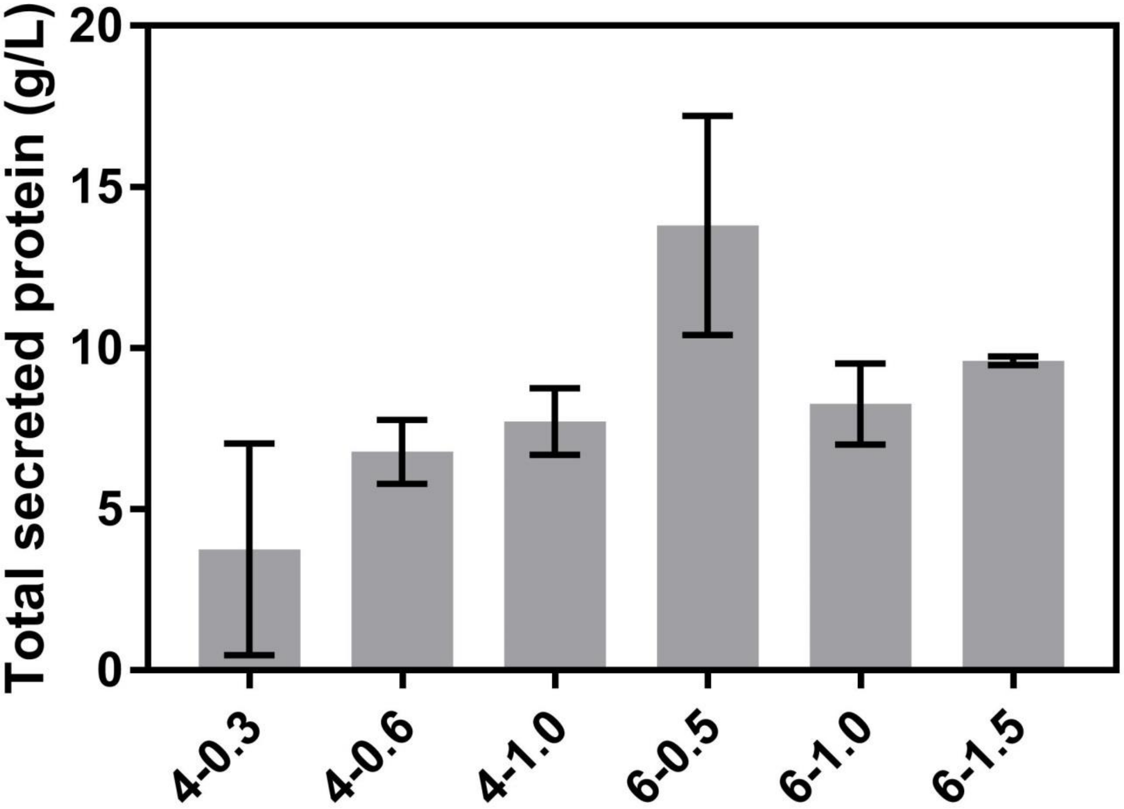

Figure S9. The yield of secretory protein by the engineered strain PD630_La after 7-days of growth on media with different carbon and nitrogen concentrations. The numbers of "X-Y" in the x-axis indicated that X% and Y% (w/v) of glucose and NH_4NO_3 were used as carbon and nitrogen source, respectively.

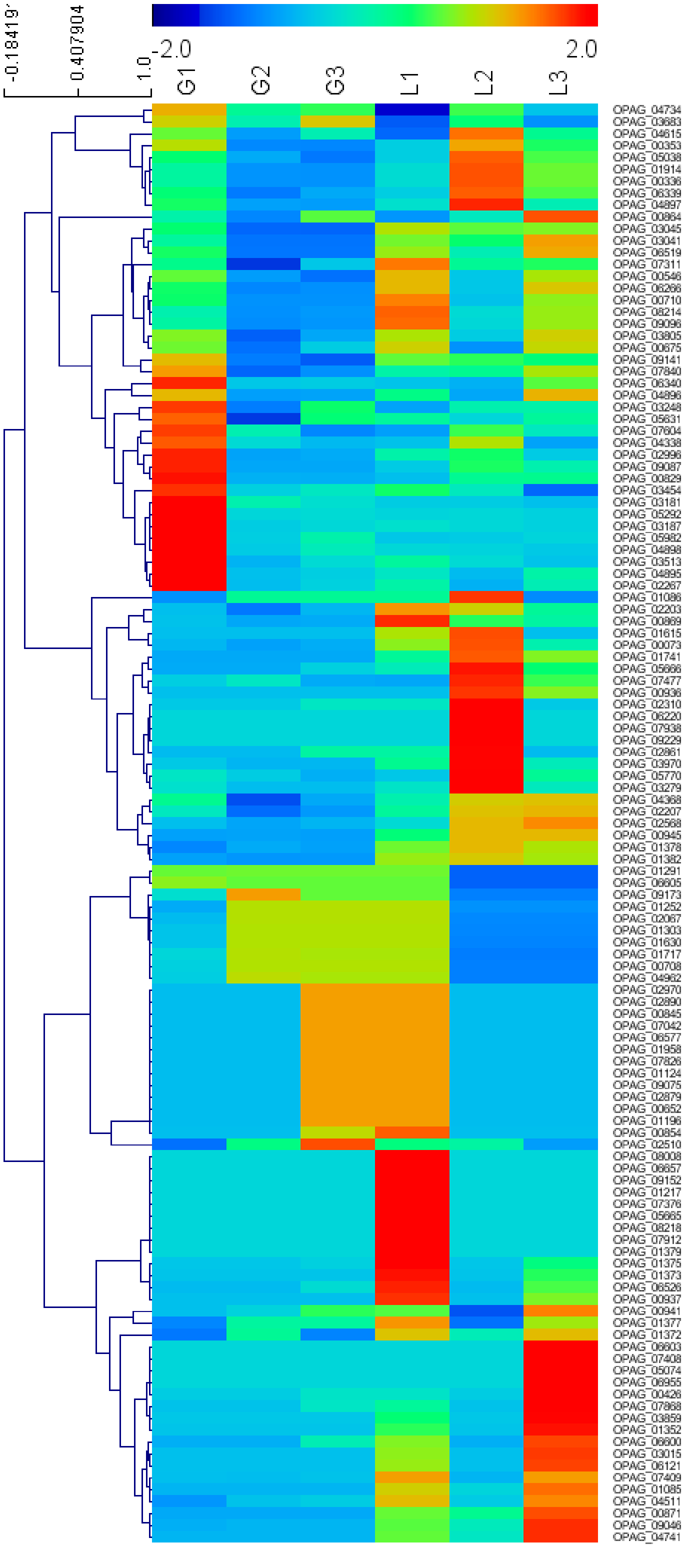

Figure S10. Hierarchical cluster of differentially expressed proteins involved in aromatic compound catabolism.

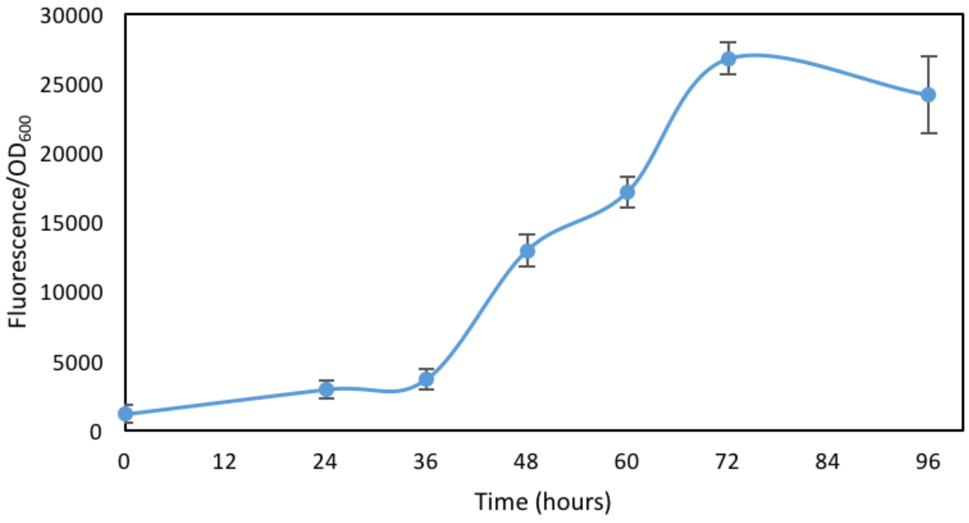

Figure S11. The lipid accumulation of *R. opacus* PD630 grown on 1% glucose. The lipid yield was indicated by Nile Red fluorescence.

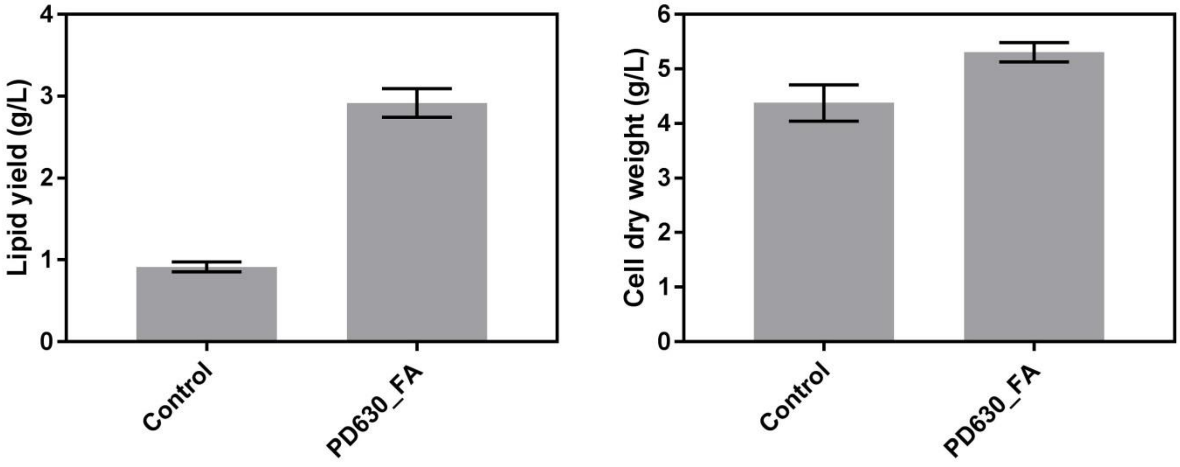

Figure S12. The comparison of lipid yield (left) and cell biomass yield (right) between the engineered strain PD630_Fa and control strain. The control strain was constructed by transformed with the same plasmid without *fasI* gene operon. 1 μg/mL thiostrepton was added at the beginning of fermentation and 2 mM sodium benzoate was added at the third day to induce the overexpression of *fasI* gene operon. The strains were harvested for biomass measuring and lipid extraction after growing on 2% glucose as carbon source with 1.4 g/L NH_4NO_3 as nitrogen source for 4 days.

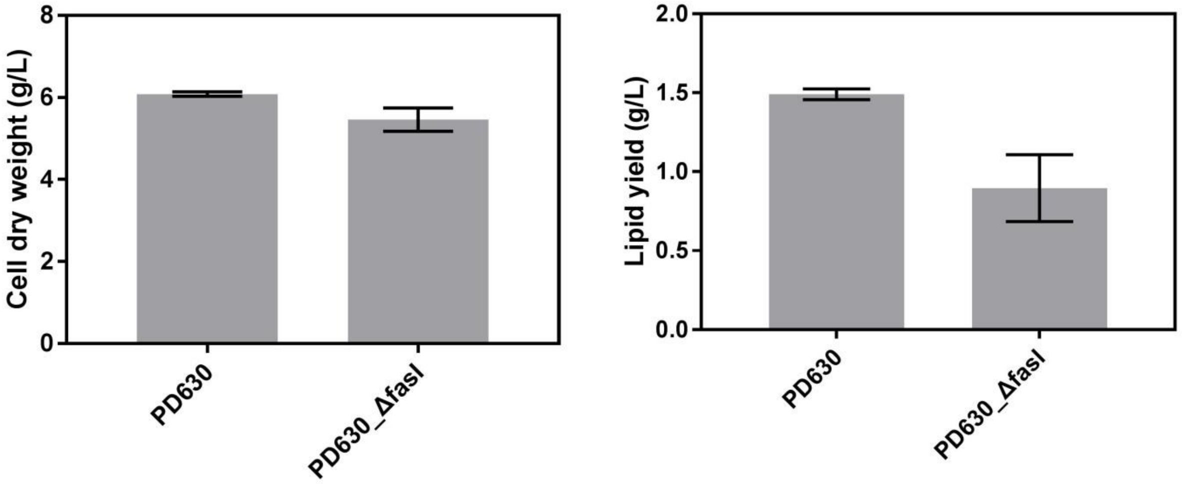

Figure S13. The comparison of cell biomass yield (left) and lipid yield (right) between the *fasI* mutant strain PD630_ΔfasI and wild type PD630. The strains were harvested for biomass measuring and lipid extraction after growing on 2% glucose as carbon source with 1.4 g/L NH_4NO_3 as nitrogen source for 4 days.

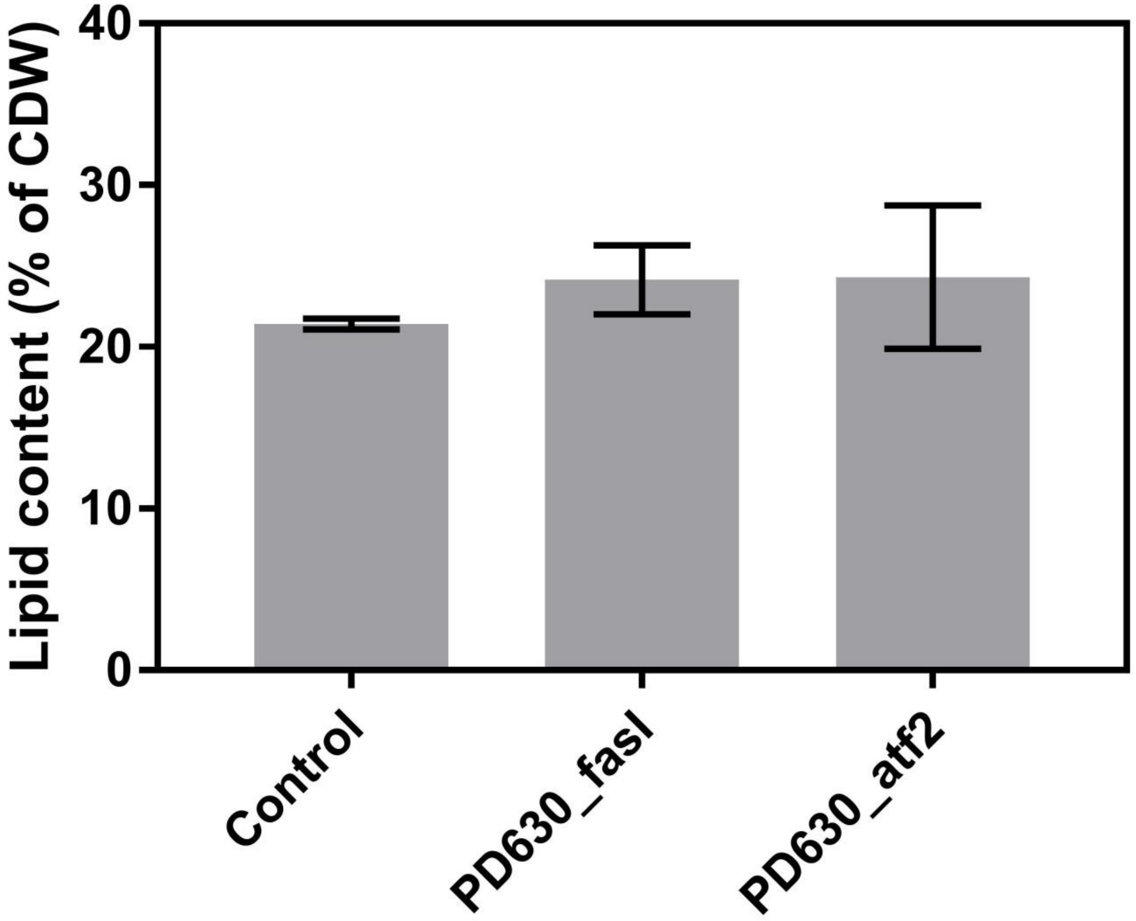

Figure S14. The lipid content of the strain with overexpression of *fasI* and *atf2,* respectively. PD630_fasI is the strain overexpression with *fasI* operon only; PD630_atf2 is the strain overexpressed with gene atf2. The control strain was constructed by transformed with the same plasmid without *fasI* gene operon and *atf2* gene. 1 μg/mL thiostrepton was added at the beginning of fermentation and 2 mM sodium benzoate was added at the third day to induce the overexpression of *fasI* gene operon. The strains were harvested for biomass measuring and lipid extraction after growing on 2% glucose as carbon source with 1.4 g/L NH_4NO_3 as nitrogen source for 4 days.

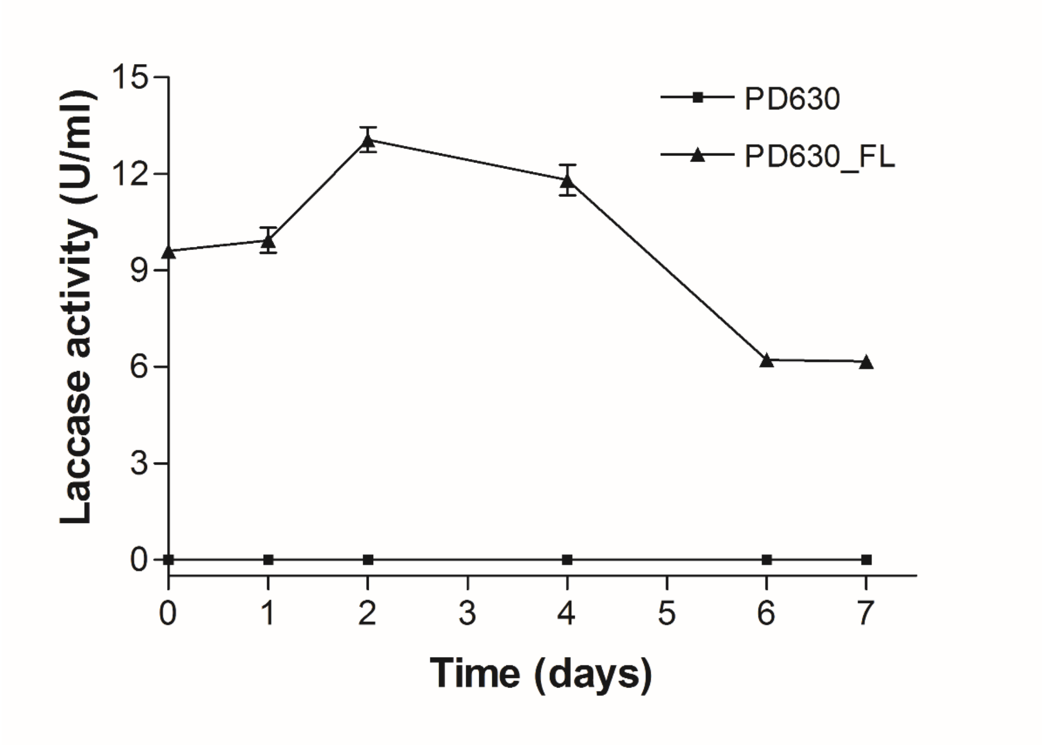

Figure S15. The laccase activity of co-transformed strain PD630_FL during fermentation on insoluble alkali lignin**.** 5 mL of pre-cultured strain from 2% glucose were inoculated to 50 mL of aforementioned minimum medium with 1% of alkali lignin as carbon source.

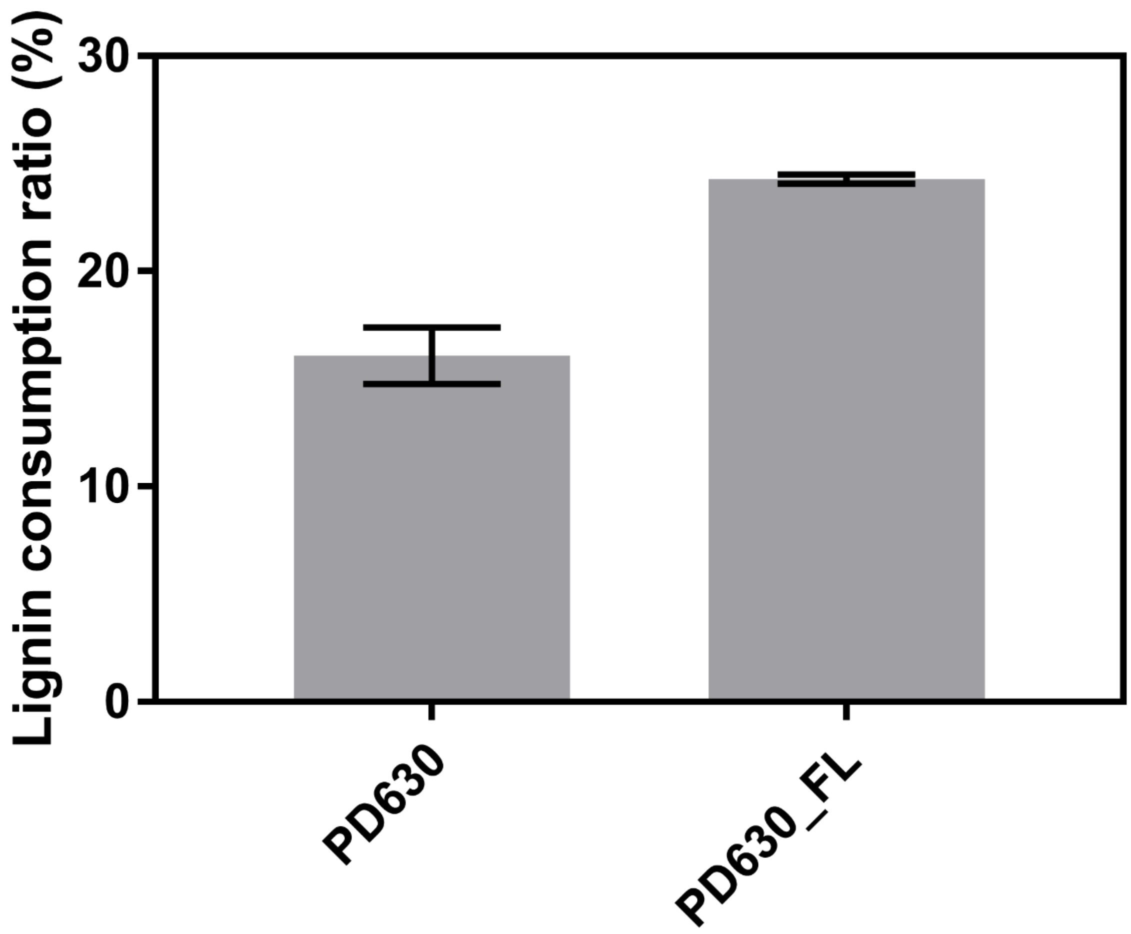

Figure S16. The lignin consumption ratio between the engineered strain PD630_FL and control strain. The lignin concentration was measured by Prussian Blue assay.

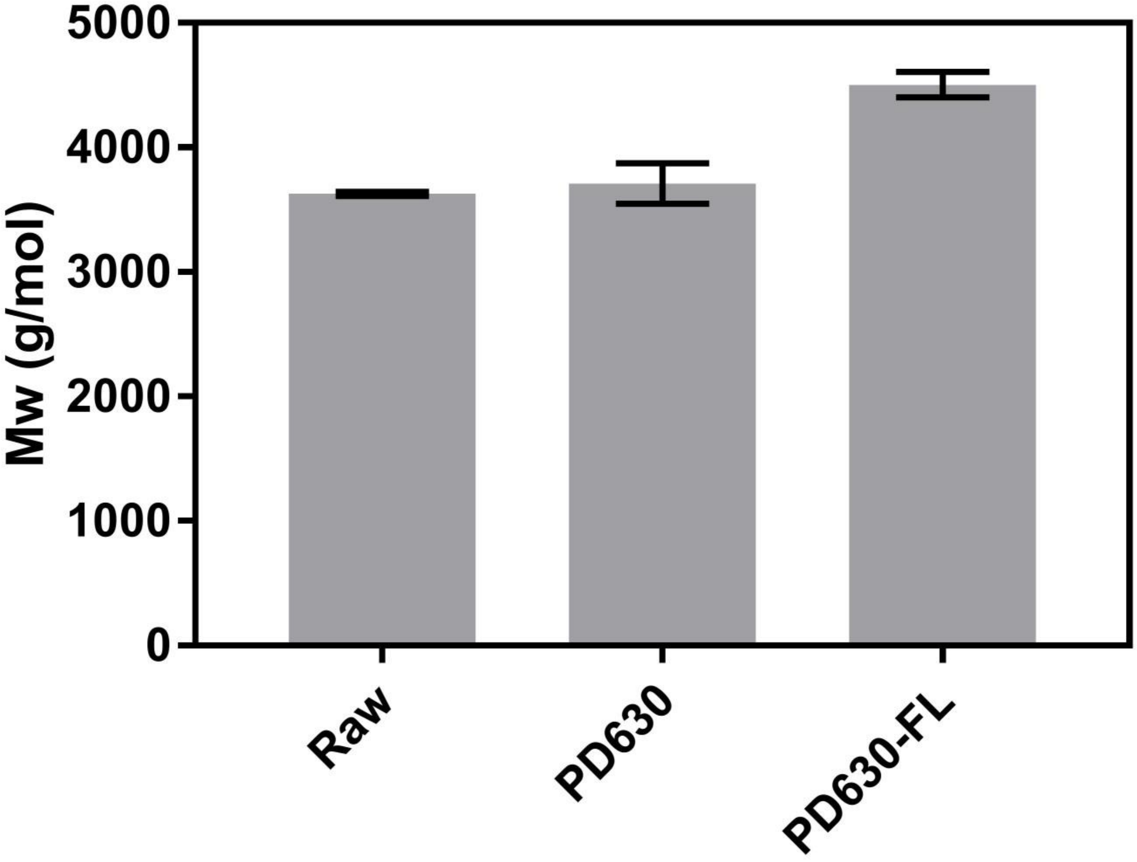

Figure S17. The molecular weight analysis of the lignin by GPC. The figure shows the Mw of the different treated lignin. Raw: raw lignin without bacterial conversion; PD630: the lignin fermented by control strain R. opacus PD630; PD630-FL: lignin fermented by engineered strain PD630_FL with laccase mediator acetosyringone.

Reference

[1] T. Matsui, H. Saeki, N. Shinzato, H. Matsuda, *Curr. Microbiol.* **2006**, *52*, 445.
[2] N. Nakashima, T. Tamura, *Appl. Environ. Microbiol.* **2004**, *70*, 5557.
[3] Y. Hong, M. K. Hondalus, *FEMS Microbiol. Lett.* **2008**, *287*, 63.
[4] R. Kalscheuer, M. Arenskötter, A. Steinbüchel, *Appl. Microbiol.Biotechnol.* **1999**, *52*, 508; K.-s. Na, K. Nagayasu, A. Kuroda, N. Takiguchi, T. Ikeda, H. Ohtake, J. Kato, *J. Biosci. Bioeng.* **2005**, *99*, 408.
[5] K. Chourey, J. Jansson, N. VerBerkmoes, M. Shah, K. L. Chavarria, L. M. Tom, E. L. Brodie, R. L. Hettich, *J. Proteome Res.* **2010**, *9*, 6615.
[6] Y. Zhang, P. Gao, Z. Xing, S. Jin, Z. Chen, L. Liu, N. Constantino, X. Wang, W. Shi, J. S. Yuan, S. Y. Dai, *Mol. Cell. Proteomics* **2013**, *12*, 3431.
[7] Y. Zhang, S. Liu, S. Dai, J. Yuan, *BMC Bioinformatics* **2012**, *13*, S8.
[8] M. P. Washburn, D. Wolters, J. R. Yates, 3rd, *Nat. Biotechnol.* **2001**, *19*, 242.
[9] J. W. Holder, J. C. Ulrich, A. C. DeBono, P. A. Godfrey, C. A. Desjardins, J. Zucker, Q. Zeng, A. L. B. Leach, I. Ghiviriga, C. Dancel, T. Abeel, D. Gevers, C. D. Kodira, B. Desany, J. P. Affourtit, B. W. Birren, A. J. Sinskey, *PLoS Genet*. **2011**, *7*, e1002219.
[10] T. Xu, S. K. Park, J. D. Venable, J. A. Wohlschlegel, J. K. Diedrich, D. Cociorva, B. Lu, L. Liao, J. Hewel, X. Han, C. C. L. Wong, B. Fonslow, C. Delahunty, Y. Gao, H. Shah, J. R. Yates Iii, *J. Proteomics.***2015**, *129*, 16
[11] T. Xu, J. Venable, S. K. Park, D. Cociorva, B. Lu, L. Liao, J. Wohlschlegel, J. Hewel, J. Yates III, "ProLuCID, a fast and sensitive tandem mass spectra-based protein identification program", presented at *Molecular & Cellular Proteomics*, **2006**.
[12] C. Zhao, S. Xie, Y. Pu, R. Zhang, F. Huang, A. J. Ragauskas, J. S. Yuan, *Green Chem.* **2016**, *18*, 1306.
[13] Y. Pu, S. Cao, A. J. Ragauskas, *Energy Environ. Sci.* **2011**, *4*, 3154.
[14] S. Xie, Q. Sun, Y. Pu, F. Lin, S. Sun, X. Wang, A. J. Ragauskas, J. S. Yuan, *ACS Sustain. Chem. Eng.* **2017**, *5*, 2215.
[15] F. Berini, M. Verce, L. Ausec, E. Rosini, F. Tonin, L. Pollegioni, I. Mandić-Mulec, *Appl. Microbiol. Biotechnol.* **2018**, *102*, 2425.
[16] S. Ma, N. Liu, H. Jia, D. Dai, J. Zang, Z. Cao, J. Dong, *J. Basic Microbiol*. **2018**, *58*, 68.
[17] X. Ma, L. Liu, Q. Li, Y. Liu, L. Yi, L. Ma, C. Zhai, *Enzym. Microb. Technol.* **2017**, *103*, 34.
[18] S. Ece, C. Lambertz, R. Fischer, U. Commandeur, *AMB Express* **2017**, *7*, 86.
[19] A. Safary, R. Moniri, M. Hamzeh-Mivehroud, S. Dastmalchi, *J. Biotechnol.* **2016**, *227*, 56.
[20] B. Wang, Y. Yan, Y. Tian, W. Zhao, Z. Li, J. Gao, R. Peng, Q. Yao, *World J.Microb. Biot.* **2016**, *32*, 40.
[21] S. Kumar, K. K. Jain, K. N. Bhardwaj, S. Chakraborty, R. C. Kuhad, *PloS One* **2015**, 10, e0144379.
[22] B. Z. Feng, P. Li, *Braz. J. Microbiol.* **2014**, *45*, 351.
[23] C. Ottoni, M. Simões, S. Fernandes, C. Santos, N. Lima, *Int. J. Env. Res. Pub. He.* **2016**, *13*, 778.
[24] M. Vrsanska, S. Voberkova, V. Langer, D. Palovcikova, A. Moulick, V. Adam, P. Kopel, *Molecules* **2016**, *21*, 1553.
[25] F. He, X. Qin, H. Zhang, Y. Yang, X. Zhang, Y. Yang, *J. Chem. Technol. Biot.* **2015**, *90*, 2265.

[26] F. Zheng, Q. An, G. Meng, X.-J. Wu, Y.-C. Dai, J. Si, B.-K. Cui, *Int. J. Biol. Macromol.* **2017**, *102*, 758.
[27] B. Bertrand, F. Martínez-Morales, R. Tinoco-Valencia, S. Rojas, L. Acosta-Urdapilleta, M. R. Trejo-Hernández, *J. Mol. Catal. B- Enzym.* **2015**, *122*, 339.
[28] O. Fokina, J. Eipper, S. Kerzenmacher, R. Fischer, *Bioresour. Technol.* **2016**, *218*, 455.
[29] R. Zhuo, P. Yuan, Y. Yang, S. Zhang, F. Ma, X. Zhang, *Biochem. Eng. J.* **2017**, *117*, 62.
[30] J. Hu, F. Wang, A. Ma, G. Zhuang, Y. Liu, J. Lu, C. Guo, C. Liu, *Eng. Life Sci.* **2016**, *16*, 364.
[31] J. Yang, T. B. Ng, J. Lin, X. Ye, *Int. J. Biol. Macromol.* **2015**, *77*, 344.
[32] P. A. Campos, L. N. Levin, S. A. Wirth, *Process Biochem.* **2016**, *51*, 895.
[33] V. Garrido-Bazán, M. Téllez-Téllez, A. Herrera-Estrella, G. Díaz-Godínez, S. Nava-Galicia, M. Á. Villalobos-López, A. Arroyo-Becerra, M. Bibbins-Martínez, *AMB Express* **2016**, *6*, 93.
[34] M. Avelar, C. Olvera, D. Aceves-Zamudio, J. L. Folch, M. Ayala, *Protein Expres. Purif.* **2017**, *136*, 14.
[35] Y. Iimura, T. Sonoki, H. Habe, *Protein Expres. Purif.* **2018**, *141*, 39.
[36] E. Balcázar-López, L. H. Méndez-Lorenzo, R. A. Batista-García, U. Esquivel-Naranjo, M. Ayala, V. V. Kumar, O. Savary, H. Cabana, A. Herrera-Estrella, J. L. Folch-Mallol, *PloS One* **2016**, *11*, e0147997.
[37] A. E. Adekunle, F. Wang, J. Hu, A. Ma, C. Guo, G. Zhuang, C.-Z. Liu, *Bioprocess. Biosyst. Eng.* **2015**, *38*, 1973.
[38] M. R. Z. Passarini, C. A. Ottoni, C. Santos, N. Lima, L. D. Sette, *AMB Express* **2015**, *5*, 19.
[39] Y. Yang, Z. Guan, Y. Ding, X. Liao, Y. Cai, *Ann. Microbiol.* **2015**, *65*, 575.
[40] A. Bronikowski, P.-L. Hagedoorn, K. Koschorreck, V. B. Urlacher, *AMB Express* **2017**, *7*, 73.
[41] P. C. Carvalho, J. S. Fischer, E. I. Chen, J. R. Yates, V. C. Barbosa, *BMC Bioinformatics* **2008**, *9*, 316.
